# Ion Mobility Mass Spectrometry (IM-MS) for Structural Biology: Insights Gained by Measuring Mass, Charge, and Collision Cross Section

**DOI:** 10.1021/acs.chemrev.2c00600

**Published:** 2023-02-24

**Authors:** Emilia Christofi, Perdita Barran

**Affiliations:** Michael Barber Centre for Collaborative Mass Spectrometry, Manchester Institute of Biotechnology, University of Manchester, Princess Street, Manchester M1 7DN, United Kingdom

## Abstract

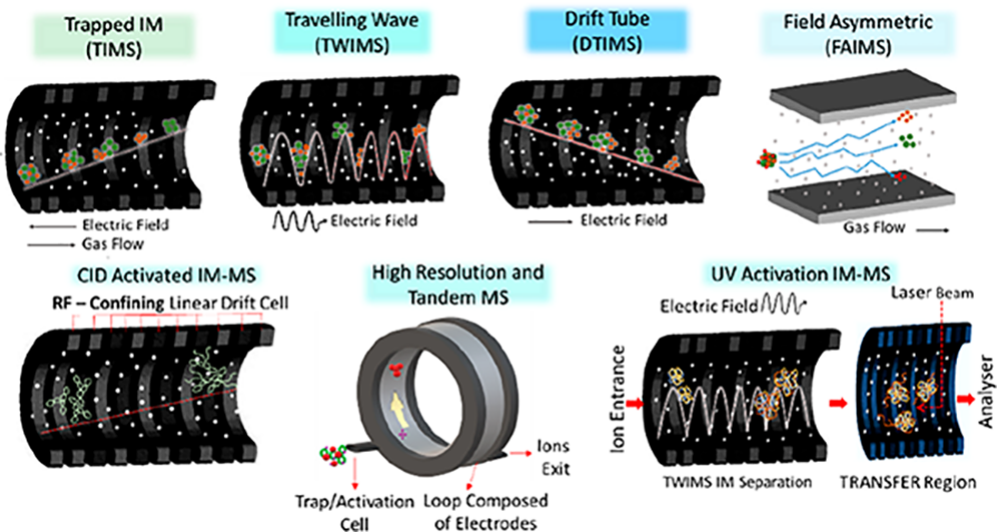

The investigation of macromolecular biomolecules with ion mobility mass spectrometry (IM-MS) techniques has provided substantial insights into the field of structural biology over the past two decades. An IM-MS workflow applied to a given target analyte provides mass, charge, and conformation, and all three of these can be used to discern structural information. While mass and charge are determined in mass spectrometry (MS), it is the addition of ion mobility that enables the separation of isomeric and isobaric ions and the direct elucidation of conformation, which has reaped huge benefits for structural biology. In this review, where we focus on the analysis of proteins and their complexes, we outline the typical features of an IM-MS experiment from the preparation of samples, the creation of ions, and their separation in different mobility and mass spectrometers. We describe the interpretation of ion mobility data in terms of protein conformation and how the data can be compared with data from other sources with the use of computational tools. The benefit of coupling mobility analysis to activation via collisions with gas or surfaces or photons photoactivation is detailed with reference to recent examples. And finally, we focus on insights afforded by IM-MS experiments when applied to the study of conformationally dynamic and intrinsically disordered proteins.

## 1 Historical Development of IM-MS

The technique of ion mobility spectrometry predates that of MS by at least 14 years. The invention of both can be traced to the Cavendish Laboratory headed by J. J. Thomson, where substantial ingenuity led to the development of many methods to measure ions.^1^ In 1894, John Zeleny went to Cambridge as an undergraduate student and performed research under Thomson’s guidance on the study of the movement of ions in gases. In a remarkable report, he showed that the velocity of atomic ions through a tube filled with a gas (he used both air, oxygen, nitrogen, and carbon dioxide) under the influence of a weak electric field was dependent on the gas and the ion.^2^ Zeleny defined the mobility (*K*) of any ion as the ratio between the velocity through which it drifts through a gas (ν_D_), and the applied static electric field (*E*). Zeleny noted that the velocity of the negatively charged ion was always (save for in acetylene) greater than that of the positively charged ion and he remarked “*We are thus led to suppose, as in liquids, that the observed velocity difference is due to an inequality in the size of the two ions. Why the two ions, even if they are formed of groups of molecules, should in a simple gas be of a different size is a question to which definite answers cannot be given in the present state of our knowledge, or rather ignorance, of the relation between matter and electricity, but is one which must be borne in mind in considerations of this relation*.” This observation is foundational to the technique of ion mobility spectrometry and remains prescient over 120 years later as it is applied to large macromolecular biological ions.

Some 14 years later, Francis Aston, again under Thompson’s guidance, reported the first mass spectrograph, and with it, evidence for the existence of isotopes.^3^ While both techniques measure ions and their movement due to the application of fields from static elements, the fundamental difference between the two is that the MS records the movement of ions through an evacuated region, whereas ion mobility is the movement through a gas. It follows that in MS Newtonian mechanics can be used to determine the relationship between transport due to the applied field and the mass of the charged particle. With ion mobility the interaction between the ion and the gas determines its passage, and therefore consideration of how the nature of the gas including its temperature, arrangement of atoms, and charge within the ion must be made to rationalize the measurement.

Despite their inceptions in the same laboratory, the coupling of MS to ion mobility spectrometry did not occur some 70 years later, when Mason and McDaniel built the first ion mobility (IM) instrumentation again to study the behaviors of principally monatomic ions in gases.^4,5^ This body of work developed the first theoretical understanding of the nature of an ion mobility measurement and gave rise to the Mason–Schamp eq 1, which relates the mobility of an ion (*K*) to its collision cross-section (Ω).^4−6^The Mason–Schamp Equation
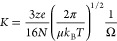
1

Equation 1: The ion mobility, *K* as calculated by the Mason–Schamp equation. The equation relates the mobility of an ion with its collision cross-section, Ω. It is evident that mobility, *K*, is inversely proportional to the collision cross-section, Ω.

The requirement for high vacuums for MS detection and resolution and elevated pressure for ion mobility means that such instruments require regions of differential pumping separating the ion source from the mobility separation and mass analysis, which presented a considerable technical challenge. The mechanism whereby gaseous protein ions are released from charged solvent droplets during electrospray ionization (ESI) remains a matter of debate. Also, it is unclear to what extent electrosprayed proteins retain their solution structure. Molecular dynamics (MD) simulations offer insights into the temporal evolution of protein systems. Surprisingly, there have been no all-atom simulations of the protein ESI process to date. The current work closes this gap by investigating the behavior of protein-containing aqueous nanodroplets that carry excess positive charge. We focus on “native ESI”, where proteins initially adopt their biologically active solution structures. ESI proceeds while the protein remains entrapped within the droplet. Protein release into the gas phase occurs upon solvent evaporation to dryness. Droplet shrinkage is accompanied by ejection of charge carriers (Na+ for the conditions chosen here), keeping the droplet at ∼85% of the Rayleigh limit throughout its life cycle. Any remaining charge carriers bind to the protein as the final solvent molecules evaporate. The outcome of these events is largely independent of the initial protein charge and the mode of charge carrier binding. ESI charge states and collision cross sections of the MD structures agree with experimental data. Our results confirm the Rayleigh/charged residue model (CRM). Field emission of excess Na^+^ plays an ancillary role by governing the net charge of the shrinking droplet. Models that envision protein ejection from the droplet are not supported. Most nascent CRM ions retain native-like conformations. For unfolded proteins, ESI likely proceeds along routes that are different from the native state mechanism explored here.^4,5,7^ These foundational studies by Mason and co-workers showed the reproducibility of ion mobility measurements, which enabled the development of standard reference measurements of the mobilities of ions in different gases, a process that continues in the present day.^5,6,8−12^ This robustness of the measurement and the relatively cheap construction costs were influential in the widespread uptake of ion mobility spectrometry as a standalone analytical tool with relevance for environmental monitoring, security,^13−19^ and healthcare applications.^20−27^ The concept of reduced mobility (*K*_0_) was also introduced to assist standardization and comparability wherein the temperature and pressure of the gas under investigation are normalized allowed measurements to be compared and instruments to be calibrated.^5,6^Reduced Mobility
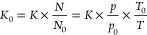
2

Equation 2: Standardizing mobility for any given ion. This relationship converts the experimental mobility (*K*) to reduced mobility (*K*_0_), which standardizes the measure for the experimental parameters of drift gas temperature (*T*) and pressure (*p*). In structural biology, reporting of ions mobility has largely been replaced by reporting of collision cross-section, CCS, values, or distributions.

The lack of a requirement for vacuum pumps meant that ion mobility spectrometers could be portable and relatively inexpensive, and since the 1970s this technology has enjoyed widespread use.^28−32^ Throughout the time that ion mobility spectrometry was gaining widespread adoption (1970s–1990s), mass spectrometers remained expensive instruments with large footprints, a requirement for ultrahigh vacuum, and there was little scientific appetite nor commercial drive to couple the two methods in a single platform.

Driven by developments in ionization allowing large atomic and molecular clusters, as well as intact biological molecules to be measured in the gas phase,^33−40^ research that coupled IM to MS started again in earnest in the late 1980s. In pioneering studies, Bowers, Jarrold, and Russell^41−45^ among others, showed the power of a technique that could provide stoichiometric and structural information for isobaric atomic clusters, as well as the dual use of such devices for gas phase ion chemistry.

The advent of the soft ionization methods of electrospray ionization (ESI) and matrix-assisted laser desorption ionization (MALDI)^37−39,45^ heralded huge developments in mass spectrometry, which were driven by the ability to now examine large molecular species and the need to optimize their transmission and detection.^46−52^ Foundational work focused on extending the *m*/*z* range of commercially available instruments because now much larger biomolecules could be placed intact into the gas phase.^48,50,53,54^ This new capability in transmission of higher *m*/*z* ions also prompted a demand for an increase in resolution because for many mass analysers resolution decreases with *m*/*z*. This was achieved by a myriad of changes to the differential pumping, ion optics design, and detection systems. The increase in the size and complexity of analytes also meant that tandem MS was more critical for the identification and structural characterization of the target analyte. In parallel, following the Human Genome Project, the rise in the use of mass spectrometry for large-scale analysis of proteins (proteomics), metabolites (metabolomics), and lipids (lipidomics), so-called ‘omics science’ led to further improvements in the rapid acquisition of data from complex samples.^55^ Much of this was assisted by increased computational capability wherein the ability to build databases of predicted biomolecules for comparison with large experimental data sets.^56−60^

Covering all of the developments in mass spectrometry from 1990 to the present day is beyond the scope of this review, however, we highlight here three examples that had pertinence for the use of MS for structural biology applications. The first was the optimization of quadrupole time-of-flight (Q-ToF) instrumentation for the transmission of large macromolecular assemblies following pioneering work by Douglas and Hunt.^48−50,53,54,61^ Modifications to a Q-ToF instrument specifically to transmit very large protein assemblies were first reported in 2002.^48^ The benefits of doing this were demonstrated with two different analytes, namely cesium iodide clusters and a heterooligomeric protein assembly formed between tetrameric transthyretin, thyroxine, retinol-binding protein, and retinol. As well as demonstrating higher *m*/*z* transmission, this work also showed how lowering the radio frequency (RF) applied to the quadrupole permits the selection of high *m*/*z* ions for tandem MS experiments. The modifications included more precise control and monitoring of all pressures in the differentially pumped regions of the instrument. They noted that for optimum transmission of large *m*/*z* ions, the instrument was operated at pressures higher than usual in every section. The installation of a leak valve to introduce argon gas between the sampling cone and hexapole (in the first stage vacuum) allowed the desolvation of ions to be optimized. This relationship between higher pressure in the first vacuum stage and improved transmission of high *m*/*z* species had been previously reported.^62,63^ Sobott et al. considered a range of sizes for the entrance and exit orifices of the collision cell to alter the velocity of the ions entering the gas-filled cell and concluded that the standard diameter of 2.25 mm was optimal. The instrument had a quadrupole that was able to isolate ions up to a *m*/*z* range between 3000 and 4000 *m*/*z*, typical for that time. By lowering the frequency of operation of the quadrupole to 300 MHz they were able to increase the *m*/*z* range that could be isolated. Cesium iodide clusters were used to demonstrate that the time-of-flight (ToF) mass analyzer could detect ions beyond 90 000 *m*/*z*. Using the RF lowered quadrupole, it was possible to isolate a single charge state of the protein complex (5340 *m*/*z*) and subject it to collisions to assess the makeup of the heteromeric complex.

A similar approach to modify a first-generation Q-ToF instrument to improve transmission of large complexes and permit a tandem MS was conducted by Van Den Heuvel et al.^50^ This modified instrument encompassed many of the same changes reported by Sobott et al.^48^ and added a few more specifically to further improve transmission and to optimize collisional activation for large complexes. The first modification was the addition of a sleeve around the first hexapole ion guide which restricted the gas flow from the source, increasing the pressure in the first part of the hexapole ion guide 3-fold. This sleeve only covers the first part, and the pressure drop on exiting this sleeved region drops linearly across the remaining 100 mm. The increased pressure in the first vacuum stage and the locally increased pressure around the hexapole entrance leads to efficient cooling of the ion beam both axially and radially, thus improving the transmission of higher *m*/*z* ions. With the lowered frequency quadrupole, they reported successful isolation of ions up to 12800 *m*/*z*. Third, they modified the collision cell to allow its operation at higher pressures by decreasing the entrance and exit orifices to 2 mm diameter. The fourth modification was the installation of high-transmission wider mesh grids in the ToF, which increased the sensitivity for large ions 3-fold. Lastly, they tuned the pusher with a lower repetition rate (410 μs) to increase the mass range. This modified Q-ToF was tested on high-mass and high-complexity macromolecules, including chaperone complexes (GroEL).^14^

Following these findings, it was clear that interest in and opportunities for studying heavier and more complex macromolecules with mass spectrometry was on the rise. In 2013, Snijder et al. were the first to report mass spectra with resolved charge-states for a 18 MDa capsid, Prohead-1^gp5^, which consists of a mixture of pentameric and hexameric capsomers and copies of the viral protease gp5.^61^ They demonstrated that 20 MDa might represent an upper mass limit for exact mass measurements on Q-ToF instruments despite claims of an “unlimited upper mass limit”. This was based on their report of the challenging process of assigning charge states to Prohead-1^gp5^ due to the broad peaks primarily resulting from poor desolvation.^61^ They used an iterative approach where they assigned and tested a wide range of charge states until the experimental mass deviated the least from the theoretical mass of Prohead-1^gp5^. This was performed across three independent replicates, with the best standard deviation being 1.3%. After this iterative and lengthy process, they concluded that maximum desolvation was required to enable resolution of individual charge-states in such MDa complexes.^61^ To demonstrate this, they prepared their sample in a dilute ammonium acetate solution to minimize the salt retention upon desolvation. They then applied the maximum voltage (400 V) to the ions passing through the collision cell filled with the heavier noble gas xenon rather than nitrogen or argon to create conditions where salt and other excipients would be collisionaly dissociated. Under these extreme conditions, they were able to resolve charge states that were normally unresolvable. These developments paved the way for future applications when ion mobility started to be commonly available in Q-ToF instruments, as we will return to later in this review.

A second notable example of a technological development that has furthered the use of mass spectrometry for biological applications is that of the orbitrap mass analyzer.^46,47^ A series of instruments that were initially introduced to study large-scale proteomics/metabolomics^64−68^ have more recently been applied for protein centric studies, both on intact denatured proteins for proteoform identification^69,70^ and for native mass spectrometry of noncovalent assemblies^71,72^ Due to the time required to obtain transients in Fourier transform mass spectrometry (FT-MS) instruments, these are technically less compatible with time dispersive IM methods, although there have been some heroic attempts^73,74^ and as such orbitraps are more commonly and much more usefully coupled to FAIMS.^75−78^

Despite difficulties in directly measuring structure of proteins with IM orbitrap MS, these instruments with their high resolving power have contributed much to the field of native MS and undoubtedly improved our understanding of protein structure. We summarize a few developments here and refer the reader to other sources for a more thorough survey of the state of the art.^79,80^ A pertinent early example is that of Rose et al., who reported the use of an Orbitrap mass analyzer to detect macromolecular assemblies using native mass spectrometry methods.^71^ Modifications to the desolvation region of the standard commercially available instrument, as well as to the RF drivers in the mass analysers, provided accurate mass measurements from a series of complex macromolecules ranging from a monoclonal immunoglobulin G (IgG) antibody (149 kDa) to an *Escherichia coli* Groel complex (901 kDa). They achieved high sensitivity (for the IgG they reported a detection limit in the range of ten attomoles, with one femtomole of sample needed for injection) as well as almost complete desolvation; compared to the Q-ToF instruments, yielding accurate mass measurements of the intact complexes. This high mass resolution allowed the antibody glycosylation profile to be resolved, demonstrating the capability of the orbitrap instrument to determine the heterogeneity of such samples.^71^

More recently, Ben-Nissan et al. utilized a more sophisticated, triple-stage mass spectrometry (MS^3^) method again on a modified orbitrap, to examine the heterogeneity of endogenous protein complexes.^81^ They applied the method to a yeast fructose-1.6-bisphosphatase 1 (FBP1) complex using a modified orbitrap mass spectrometer which permitted ion trapping in the inject flatapole.^81^ In their workflow, the intact FBP1 complex is first transmitted intact to establish the sample heterogeneity (MS^1^), which revealed the phosphorylation of the complex at up to four different sites as a result of glycolysis. Then a given FBP1 proteoform was isolated and subsequently fragmented down to its subunits using the inject-flatapole modification (MS^2^), which led to monophosphorylated units. Lastly, these subunits were further fragmented in the high-energy collision dissociation cell (HCD) to determine the site(s) of the post- phosphorylation (MS^3^), revealing two mutually exclusive modification sites.

Developments in mass spectrometry and in particular within instruments coupled to ESI sources, were also assisted by the widescale application of RF confining ion funnels and stacked ring ion guides,^51,82,83^ which minimized ion loss and allowed dilute protein complexes to be detected. Such ion funnels and guides, which we highlight as a third crucial development over the past 20 years, also heralded a new era for IM-MS, and one with great applicability for structural biology.^84−90^ In this review, we will cover the main components of modern ion mobility mass spectrometers as applied to biological macromolecules. We will first consider ionization techniques and then describe the most common ion mobility devices as well as the MS techniques that can be used alongside the mobility measurement to probe structure and stability.

One of the most attractive aspects of IM-MS measurements is that collision cross section (CCS) values can be predicted.^91−99^ It is obvious that knowledge of the sequence of a given protein will allow its mass to be predicted, but less widely known than a similar process that can be performed for its CCS if there is a comparative structure. We will describe the common methods available for doing this as well as examples of use. Understanding the nature of the measurement means it is possible to enumerate the CCS of a given ion from starting coordinates (either theoretical or obtained from other structural methods) such that its conformation can be measured and compared to candidate geometries. In this review, examples of the use of IM-MS as applied to structural biology will illustrate the capability of this method.

## 2 Methodology to Perform IM-MS Measurements for Structural Biology

### 2.1 Introduction to Biological MS Methods in the 21st Century.

Several important technological developments have positioned MS in the 21st century as an analytical tool with high versatility to identify and quantify biological macromolecules, including proteins, lipids, and metabolites.^8,12,100−109^ In the investigations of proteins, much emphasis has been on so-called “bottom-up” or “peptide centric” methods wherein proteins, often from complex biofluids, are enzymatically digested into peptides before MS analysis, and the ensuing data is then compared to that predicted from genomic databases.^55,110,111^ These approaches have enormous predictive power and have been used to provide insight into many aspects of biology, health, and disease.^67,69,112−117^ The experimental workflow destroys the functional form of the proteins under investigation, removing all higher-order structures and leaving this to be evaluated in silico and interpretation. By contrast, structural MS or native MS approaches often start with purified complex complexes and seek to retain noncovalent interactions that are responsible for the functional form of the biological complex(s) under investigation.^41,72,80,85,118−121^ In this review, we will focus on the development and application of IM-MS to determine the stability, structure, and dynamics of intact proteins and their complexes.

### 2.2 Ionization for Native MS: Electrospray Ionization (ESI), Nano-Electrospray Ionization (nano-ESI), and the Production of a Charge State Distribution (CSD)

Common to all MS analyses is the requirement for the analytes to be ionized. Structural MS investigations rely on soft ionization sources, which primarily are based on either ESI or MALDI. While MALDI has been extensively used over the past few decades, and even for intact protein analysis,^41,118,122,123^ here we focus on ESI,^37,39,124^ which is much more extensively applied within native MS, wherein the analyte is transferred directly from solution into a desolvated form in the source of the mass spectrometer.

Most native MS studies employ a variant of ESI that uses narrow bore emitters, called nanoelectrospray ionization technique (nano-ESI) with nL/min flow rate compared to μL/min in conventional ESI.^125,126^ A combination of lower flowrates and narrow bore emitters results in droplets of smaller diameters; 50–100 μm for conventional ESI and 0.5–10 μm for nano-ESI).^125,127^ Due to the difference in the initial droplet size, nano-ESI droplets require fewer evaporation/fission cycles before the analyte is fully transferred to the gas phase.^126,128−130^ The significantly smaller droplet size reduces the possibility of more than two species being present in a droplet at once and hence minimize interferences from salts and other contaminants.^126,129,131,132^ Juraschek et al. investigated this by spraying a 10^–5^ M insulin/10^–2^ M NaCl sample using a nano-ESI source.^126^ Although Na adducts were visible, the spectrum was dominated by insulin ions, and sodiated insulin ions only rose in intensity once the NaCl molarity was increased by a fold. Using the NaCl–insulin cluster system, Wilm et al. demonstrated that the possibility that a single insulin molecule and a single NaCl molecule could co-occupy a single droplet would be 1:1000, on the basis that the desolvated droplet is approximately 1/1000 of the volume of the initial droplet.^131^ These examples and many others^126,127,132−140^ all indicate that nano-ESI tolerates a higher concentration of nonvolatile salts and other small molecule excipients in the sample solution than conventional ESI. Overall, compared to ESI, nano-ESI has lower sample consumption, relatively higher nonvolatile salt concentration tolerance, and higher sensitivity.

**Figure 1:**
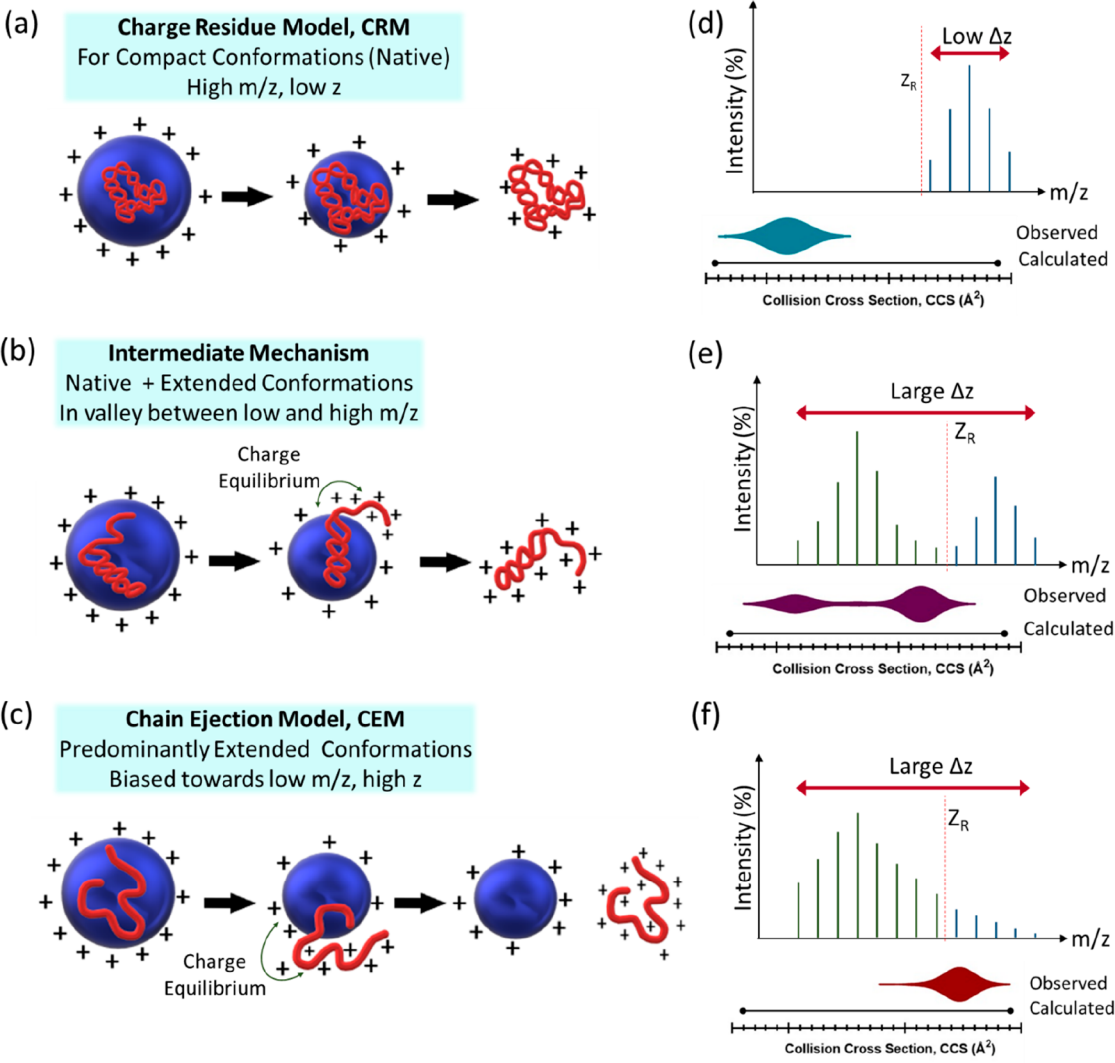
Electrospray ionization (ESI) mechanisms. (a) Charge residue model (CRM): charge deposition on the analyte following solvent evaporation, proposed for globular intact protein (b) intermediate mechanism. As proposed by Beveridge et al.,^144,145^ there is an initial ejection of the extended domain followed by the structured domain, suggested for proteins with native and extended domains and (c) chain ejection model (CEM), initial ejection of the extended chain from the droplet, proposed for unfolder protein and distorted polymer chains. Proton equilibrium occurs between the droplet surface and the protein chains in mechanisms (b) and (c). (d) Charge state distribution (CSD) of a compact conformation, under the Rayleigh limit (*Z*_R_), translating to smaller CCS values. (e) Bimodal CSD of a conformation with both structured and extended domains, overcoming the Rayleigh limit, Z_R_, and covering a wider range of CCS values, and (f) unimodal CSD of an extended conformation overcoming the Rayleigh limit, *Z*_R_, and mainly dominating the higher CCS values. ESI mechanisms. Adapted with permission from ref (144). Copyright 2015 Wiley-VCH.

There are several mechanisms proposed for the process whereby analytes exit the solvated state. The first one is the charge residue model (CRM), which is generally considered the mechanism for globular structured proteins, and the chain ejection model (CEM), which is proposed for extended proteins with higher *m*/*z* proteins and other polymers (Figure 1).^129^ ESI analyte desolvation and charging mechanisms have been extensively reviewed,^7,127,131,140−143^ and here we will provide a brief summary. During the CEM (Figure 1c), an extended protein/polymer migrates to the liquid–vapor interphase where initially one of the termini is expelled to the gas phase.^129,144^ The remaining chain undergoes stepwise ejection while undergoing charge equilibrium until it fully separates from the droplet. In the CRM (Figure 1a), the solvent droplet undergoes steady evaporation of the solvent, and in the last stages, the remaining charged analyte is left dried.^129,144^ In the study of conformationally dynamic complexes, and in consideration of commonly observed bimodal or multimodal charge state distributions, it is pertinent to add a third mechanism, which is an intermediate between the CRM and CEM proposed by Konermann (Figure 1). As outlined by Beveridge et al., intrinsically disordered proteins (IDPs) can be found in a range of conformations ranging from compact to extended to species comprising both compact and extended domains.^144−146^ Similar conformational landscapes are found for other proteins under conditions when the solution pH is more than one unit away from the isoelectric point (pI). When considering how a molecule of such a complex conformation is transferred to the gas phase, it is instructive to consider three different levels of structure, compact, intermediate, and extended. The intermediate state may be a partially unfolded form where either or both termini are no longer in the native fold, or representative of the native form where some of the protein is folded and some intrinsically disordered.^145^ In either case, the more extended part of the protein will contain a higher number of charges. Beveridge et al. proposed a mechanism of ESI desolvation for such partially compact, partially extended forms (Figure 1b). In this, the extended and more charged region of sequence will be more likely to migrate to the droplet’s surface due to its larger hydrodynamic radius, where it can be ejected akin to the CEM and will either drag through the globular part or stall as the more globular and lower net charge/mass part is desovlated via the CRM. All three mechanisms can progress simultaneously during the ESI process depending on the conformation of the isolated biomolecules, and the ensuing charge state distribution will therefore reflect the proteins’ conformational diversity in the solution. Where the charge state distribution (CSD) is narrow and high *m*/*z*, low *z* (Figure 1d), the CRM will dominate; where the charge state distribution is wide and biased toward low *m*/*z*, high *z* (Figure 1f), the CEM will dominate and where there are ions that are in the valley between low *m*/*z* and high *m*/*z*, it is likely that the intermediate mechanism is involved (Figure 1e). It is also worth noting that the source pressures can effect desolvation and alter such distributions.^63^

The stability of charges in spherical objects was first described by Rayleigh and has been used to explain the fission processes in ESI droplets as well as the presentation of charge states on proteins in the ESI spectra.^147^ The process is a function of two forces: the surface tension (keeps the droplet spherical) on the droplet’s surface and the Coulomb force of repulsion (spreads the charge evenly around the droplet) (eq 3). When in equilibrium, the droplet retains its spherical form. Once a droplet reaches the Rayleigh limit locally, the Coulomb forces overcome the surface tension forces and it undergoes Taylor cone fission^131^ to lower the charge density of the primary droplet and regain its stable spherical form.^127^ In ESI, this process is repeated until the analyte becomes a gas-phase ion.Rayleigh Limit
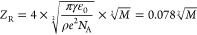
3

Equation 3: In the Rayleigh limit equation, γ is the solvent surface tension, ρ is the water density, ε_0_ is the permittivity of the surrounding medium, *N*_A_ is the Avogadro’s number, *M* is the molecular weight of the protein, and *Z* is the charge number.

De La Mora applied the Rayleigh model to explain the distribution of charge states in ESI mass spectra of proteins, and was able to discern an empirical relationship that provides the limit to the maximum charge that a spherical protein could hold (eq 4):^143^De la Mora Empirical Relationship
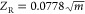
4

Equation 4: In the De la Mora empirical relationship, *Z*_R_ is the maximum charge a spherical protein can hold, and *m* is the mass of the protein under consideration.

The median charge states of folded proteins under physiological-like conditions lie under this line, whereas the majority found for unstructured proteins lie above it, in line with the assertions made for ESI desolvation mechanisms above (Figure 1). The De La Mora relationship, commonly used to justify folded/compact low charge state species, can also be used to provide a boundary between predominantly or completely folded/globular proteins and unfolded/extended proteins (see Figure 1d–f, where the Rayleigh limit is indicated) and has high utility in describing the outputs from native MS, and even more so in conjunction with ion mobility data.^10,148−150^

## 3 Developments in Ion Mobility Mass Spectrometry (IM-MS) Instrumentation and Their Implementation for Structural Biology

Ion mobility spectrometry is based on measuring the mobility of ions as they travel through a region filled with gas under the influence of electric fields.^151,152^ This mobility can be converted to a CCS, and as such it measures the structure of a gas phase ionic analyte. While as noted above, IM is often used as a standalone method, it can also be combined with mass spectrometry, and this hyphenated method has recently been widely applied for structural biology^84−89,99^ and is the focus of this review.

In order to understand the opportunities offered by IM-MS for structural biology above that offered by mass spectrometry alone, it is important to consider how each charge state can comprise a different conformation or even several conformations of the biopolymer under investigation. For proteins, as discussed in section 2.2 above, ions with higher charge states present with higher experimental CCS and correspondingly lower arrival times, as predicted by De la Mora.^143^ The Rayleigh limit as described by eq 3 enumerates how Coulombic effects drive the unfolding of proteins. If two protons are close in the desolvated protein, they will repel each another and this will drive an unstructuring process.^147^ This often corresponds to a minimum in a charge state distribution, dividing charge states that correspond to the natively folded or compact form of the protein and more extended and denatured forms. While such reading of charge state distributions can be interpreted to determine the folded state of any given protein,^153,154^ IM-MS measurement will provide a more direct readout of the conformations adopted. The distinct advantage of an IM-MS workflow over an MS workflow is the ability to determine unfolding intermediates often in the low intensity region of a charge state distribution and in single charge states.^155^

**Figure 2:**
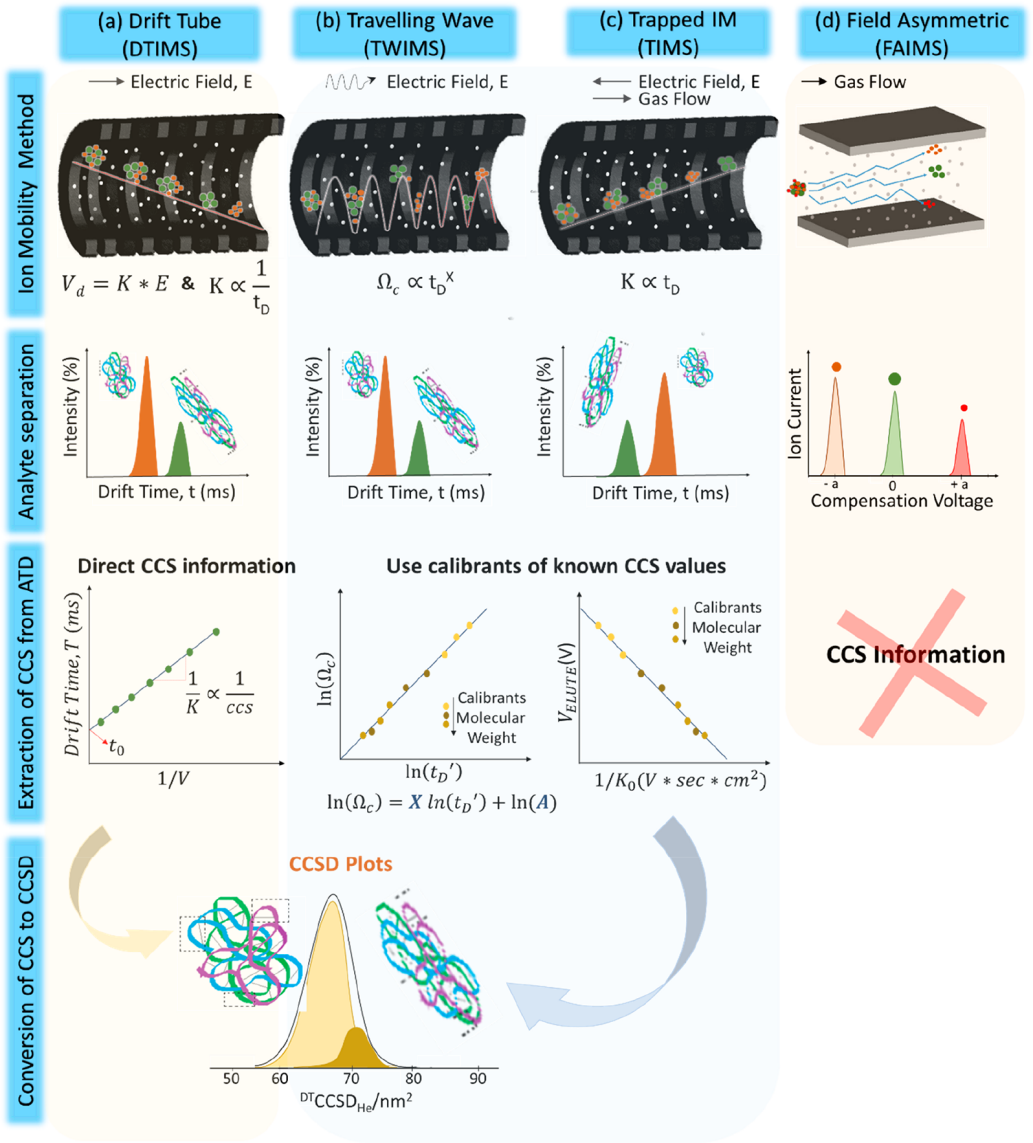
Ion mobility mass spectrometry (IM-MS) methods and the extraction of collision cross section distributions (CCS) distributions from arrival time distributions (ATD). (a) Drift tube ion mobility spectrometry (DTIMS), where a static cocurrent electric field is applied across the drift cell which contains gas. Commonly, measurements are made under different field strengths by stepping the applied field. (b) Traveling wave ion mobility mass spectrometry (TWIMS) with a rf confining field to a stacked ring ion guide and an imposed traveling wave of a given low voltage pulse applied cocurrent across the drift cell which contains gas. (c) Trapped ion mobility spectrometry (TIMS) with cocurrent gas flow and countercurrent electric field applied. (d) Field asymmetric ion mobility spectrometry (FAIMS) with cocurrent gas flow and variable intensity asymmetric waveform electric field applied. Here for a given applied waveform, a compensation voltage is applied to transmit ions of a given mobility (green dot) and exclude ions of different mobilities (red and orange dots). For DTIMS, collision cross-section (CCS) are obtained using the Mason–Schamp equation by plotting drift times (*t*_D_) that arise from different fields against the inverse of the voltage (*V*) (a). The slope of the line is the inverse of the ion’s mobility (*K*), which in turn is proportional to the inverse of the CCS. The time the ions spend outside the mobility cell is termed *t*_0_. TWIMS and TIMS require calibration with analytes of known CCS values for the extraction of the CCS data (b,c). For TWIMS, the collision cross sections Ω_c_ are plotted against the corrected drift time *t*_D_′ (b). The regression parameters *A* and *X* are then used to derive the CCS. For TIMS the *V*_ELUTE_ is plotted against the reduced motilities of known calibrants (c). FAIMS does not readily provide CCS information (d). Each method involves a buffer gas (white dots), commonly nitrogen or helium. TWIMS, TIMS, and DTIMS, all involve a cell of similar configuration through which the ions drift (a–c). FAIMS has a substantially different geometry (d). The applied electric field in each case differs, but in common, all measure the mobility of ions in the presence of an electric field and a gas.

IM analysis of biomolecules for their structure has, to date, primarily been performed in one of two instrument configurations: drift tubes (DT)^42,156^ or traveling wave IM separators (TWIMS).^84^ Other methods not as extensively used for native MS studies are trapped IM (TIMS)^157^ and field asymmetric IM (FAIMS).^158^ All four versions of ion mobility are illustrated in Figure 2 with reference to their general mode of operation and capabilities. FAIMS can be used to separate analytes using a compensation voltage but cannot provide the user with CCS information.

### 3.1 Drift Tube Ion Mobility Spectrometry (DTIMS)

In DTIMS, which is the form first used by Zeleny,^2^ (Figure 2a), a pulse of ions is injected into a tube of a given length (d) containing an inert buffer gas across which is applied a potential (*V*). The mobility (K) of an ion is the proportionality constant between the ratio of the drift velocity (*V*_d_) and the applied field, *E*(*V*_d_/*E*). As long as the length (*d*) of the drift tube is known, *K* can be readily obtained by measuring the arrival time of the ions. A common workflow is to record the ions’ drift time at a range of potentials (*V*) (Figure 2c).

In such IM instruments, ions are separated according to their *m*/*z*, their shape, and their interactions with the chosen buffer gas at the experimental temperature. The shape is, in fact, a composite of the drag experienced by the ion due to collisions with the buffer gas and the interactions that each buffer gas atom/molecule has with the analyte.^6,91^ The mobility of the ion can be converted to a CCS using the Mason–Schamp equation, which approximates the momentum transferred on collision to the first integral.^4,5^ In general, compact structures with smaller surface areas undergo fewer collisions with the background gas and present with smaller DT whereas elongated structures have longer DT, although ions can also be resolved due to the distribution or location of available charge^95,159^ and a heavy ion with the same geometrical radius as a lighter one will have a larger CCS.^91^ Another important point to note is the relationship between temperature and CCS because the computed CCS is a function of the effective temperature of the ion and the ion neutral interaction potential.^156^

The resolution of DTIMS systems (*R*_DT_), is given by the ratio between the drift time (*t*_d_) and the width the peak (Δ*t*) (equation 5). For a rigid body it is dependent on several instrument parameters including the width of the injection pulse, the length of the tube, the voltage-drop (Δ*V*) across the drift cell, and the buffer gas and effective ion temperature (*T*) and the gas pressure (*P*).^42,51,156,160−163^ To remain within the low-field limit,^5^ such that ions do not align with the field, any increase in pressure (*P*) must be accompanied by an increase in voltage (*V*), but this is not practical as even for inert buffer gases electrical discharges can occur.^42^ Therefore, to increase the resolution (*R*_DT_) either the temperature (*T*) should be reduced, or the length of the drift cell should be increased (eq 5). Heroic attempts have been made to develop very long linear field drift tubes as well as the development of circular linear field systems.^164−170^ Both are hard to translate to a commercial offering, however high-resolution linear drift field IM-MS has been realized by Agilent^171,172^ and circular geometry high resolution IM-MS by Waters (see below).^165,173^Resolution of DTIMS System
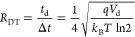
5

Equation 5: In the equation for the resolution of DTIMS systems, *t*_D_, is the drift time of the gas phase ion (apex of the ATD), Δ*t* is the time difference or width of the ATD. *K*_B_ is the Boltzmann constant, *T* is the gas temperature, *V*_d_ is the drift velocity, and *q* is the charge of the analyte ion

The use of DT IM-MS to measure the change in CCS as a function of charge on a protein following ESI represented the first examples of how this method could be used to examine protein structure and dynamics in work performed more than 20 years ago.^85,151,169,174−176^ Such studies are of course not limited to DT IM-MS instrumentation (see below) and following pioneering work by Jarrold and co-workers,^177^ it is now widely accepted that unfolded proteins will have higher numbers of protonated sites and commensurately higher CCS values.^143,178−180^

### 3.2 Traveling Wave Ion Mobility Spectrometry (TWIMS)

Unlike DTIMS where a static electric field is applied for analyte separation, TWIMS uses a traveling wave potential (Figure 2b),^84,90,181^ applied across an RF confining drift cell. The wave-shaped potential consists of a series of transient direct current (DC) voltage, which is superimposed on top of the RF voltage. TWIMS separation follows the same concepts as DTIMS where; expanded conformers surfing the wave, have a higher surface area available for collisions with the inert gases in the stack resulting in delayed arrivals to the detector. However, unlike DTIMS, TWIMS does not provide direct information on the CCS of the analytes from first principles^10,182^ but instead requires calibration using standards of known CCS values.^10,183,184^ More on the calibration approach is discussed in section 3.5.

### 3.3 Trapped Ion Mobility Mass Spectrometry (TIMS)

TIMS differs from the other two techniques DTIMS and TWIMS, because the ions are met with an electric field of counter flow to that of the ion and a cocurrent gas flow (Figure 2c).^157,160,185−187^ Together, the cocurrent gas flow and countercurrent electric field, work to separate ions according to their mobility. The countercurrent electric field keeps the ions stationary, and the cocurrent gas pushes the ions through the TIMS analyzer. With the use of an electrical gradient, ions with different mobilities are trapped at different potentials. In an opposite manner to TWIMS and DTIMS, ions of higher surface area will be trapped later in the cell compared to smaller surface area ions and will be eluted first by lowering the applied voltage intensity (Figure 2c). In terms of operation, separation of ions of differing mobilities with TIMS can be thought to be the inverse of what occurs with DTIMS.

Obtaining CCS values from TIMS, ATDs requires calibration from proteins of known mobilities.^187−190^ There are three ways to calibrate TIMS data. Hernandez et al. published a method where the inverse of the reduced mobility (*K*_0_) is plotted against the voltages applied to elute the analytes after trapping (*V*_ELUTE_)),^188^ (2) Michelmann et al. uses a plot of the inverse *V*_ELUTE_ against known reduced mobility values from literature.^187^ The most recent calibration method was developed by Chai et al., who use a sample-independent calibration method modeled by the Taylor expansion series derived from a Boltzmann transport equation.^189^ The latter is transferable as with a single mathematical equation and a set of parameters one can calibrate their mobilities for any instrument settings. The Hernandez et al. and Michelmann et al. methods, although based on different approaches produce equally precise *R*^2^ and *y*-intercept values. The *y*-intersection point on the plots is the voltage applied to the exit funnel of the instrument. The choice of calibration method should be kept consistent in comparative experiments.

### 3.4 Field Asymmetric Ion Mobility Spectrometry (FAIMS)

In this review, we will only describe the general operation of FAIMS to form a complete picture of the types of IM techniques available. FAIMS has a substantially different geometry and similarly to TIMS requires a cocurrent gas flow (Figure 2d). Also called a filtering device, it makes use of low and high wave-formed electric fields to separate the ions.^6,158,160^ Only ions responding to the varied field and matching the compensation voltage applied can be successfully separated, all other ions are simply ejected from the cell. The biggest limitation of this technique compared to DTIMS, TWIMS, and TIMS is its incapability to readily provide CCS data, however, as mentioned it has substantial benefits acting as a filter when sampling from complex mixtures^76,77,191^ and may well be applied widely in the future on triple quad mass spectrometers.

### 3.5 Obtaining CCS Values from Experimental Data

The primary product from all IM-MS experiments is the arrival time distribution (ATD) of an analyte. To gain more insightful information on the structure of the molecules, the ATD can be converted to a distribution of the CCS values measured.^10,182^ As mentioned in detail (sections 3.1, 3.2) DT IM-MS does not require calibration with known proteins’ CCS values (Figure 2a). Hence, in this section, we will only consider the case of TWIMS where calibration is mandatory to convert the ATD to CCS distributions using known calibrants (Figure 2b). Detailed protocols for the calibration of TWIMS data using known calibrants are extensively available in literature.^10,183,192−195^ With the TWIMS approach, the inverse relationship between CCS and drift time is not valid due to a nonstatic field being used, and thus the CCS cannot be calculated simply by using the Mason–Schamp equation (eq 1).^194^

When choosing calibrants, one must ensure that they cover a range of *m*/*z* values (“window”) around the targeted analyte and that they are of the same molecular type, if possible.^6,10,183,194^ Various studies have reported the CCS values obtained from proteins and other biomolecules that are readily available, although to date there is no single database that acts as a comprehensive source of CCS data.^183,196^ It is always good practice to iterate tuning conditions for the target analyte and then run the calibrant compounds under those conditions in triplicate. Due to the difficulties in preserving native conformations, a suggested method is to use native standards to assist tuning for the target analyte and to calibrate with denatured proteins because their CCS values are dictated by charge effects and are more reliable.^10^ Target sample and calibration data should be acquired across different days to account for variations in the drift time due to changes in instrument and laboratory conditions. This experimental approach ensures data reproducibility and decreased error.

Common practice in TWIM instrumentation is to record the ATD of calibrant proteins and then correct for their charge, reduced mass, and time spent outside the drift cell after the IM separation.^194^ A logarithmic plot of the experimentally corrected drift times ln(*t*_D_′) in the *x*-axis against the ln(CCS (Ω_c_)) in the *y*-axis (Figure 2b) is then produced. The calibration curve can also be displayed in a linear fit of *t*_D_′ vs Ω_c_ and in either fitting should only be considered for R^2^ ≥ 0.98. If the calibration curve meets the *R*^2^ criteria, the exponential factor (*X*) and fit-determinant constant (*A*) are extracted and used for the conversion of ATDs to CCS. To make the CCS of different charge states comparable and plot them on a single graph, the last step is to scale them against the base peak in terms of both MS intensity and area under the peak. These considerations help convert individual charge state’s CCS to CCS distribution plots.^10^

Calculating CCS from calibrant proteins by hand involves long hours of manual data manipulation. In recent years, methods to automize these calculations have started to be released. An example is AutoCCS, which is Python-based software enabling automatic CCS calculations with minimal manual input.^197^

### 3.6 Commercially Available IM-MS Platforms

As described in sections 3–3.4, there are several different ion mobility configurations that can be coupled to MS which vary in resolution, ion transmission characteristics, and sensitivity as well as the suitability for a given application area.^6,75,158,160,172,198,199^ The most prevalent type of mass spectrometer that is coupled to the time dispersive forms of IM (DT, TWIMS) and the confinement and selective release forms (trapped IMS) is a quadrupole time-of-flight (Q-ToF). This is primarily due to the ability to take many ToF spectra (typically at a frequency of μs) for each arrival time distribution (typically 0.8–10 ms peak width). The manufacturer names for these Q-ToF based systems as Agilent 6560 IM-Q-TOF,^172,200^ Waters Select Series Cyclic IMS,^165^ Waters SYNAPTs G2, G2S, G2Si, XS,^84,201^ timsTOF HT.^157,185^ and FAIMS, which is a spatially dispersive IM method, has also been coupled to Q-ToF instruments^202,203^ but is more conventionally coupled to Orbitraps or to FT-ICR.^75,77^

The structures for lossless ion manipulation (SLIM), technology was first developed by Ibrahim, Smith, and co-workers.^204−206^ MOBILion SYSTEMS has collaborated with Agilent to mount SLIM technology on a Q-TOF, and this configuration has been available in the market since June 2021. In Table 1, we list common commercially available IM-MS platforms. We have indicated the IM resolving powers of such IM technologies along with the individual advantages and disadvantages. We anticipate that with ongoing instrumentation developments and modifications, the values captured in this table will alter.

**Table 1:** Commercially Available Ion Mobility (IM) Technologies (as of 2022) along with the Mass Spectrometry Platforms to which They Are Coupled^a^

	drift cell dimensions	type of mass spectrometer	common product name(s)	IM resolving power	how to determine CCS from mobility	advantages	disadvantages
linear field drift cell (DT)	5–100 cm	Q-TOF	Agilent 6560 IM-Q-TOF	∼50–60	direct CCS information, from first principles	CCS values can be measured and calculated from first principles, Mason–Schamp	ions are pulsed, decreasing the duty cycle (trapping time/separation time DT)
traveling wave cell (TW)	∼20–40 cm	Q-TOF	Waters Synapt G1, G2, G2S, G2Si, XS Vion	∼30–40	requires calibration from known CCS values	ability to manipulate ion motion into long path length separations with no significant ion loss	requirements for calibration, ion heating on injection
cyclic (cIM)	dependent on passes (each equals ∼98 cm)	Q-TOF	Waters cyclic HDMS select ion series	∼60–80 **(One pass)** ∼750 **(100+ passes)**	requires calibration from known CCS values	cyclic loop provides extremely high resolution and is capable of tandem MS	ion transmission reduces with increasing passes
trapped (TIM)	∼1 m	Q-TOF	Bruker	200–400	requires calibration from known CCS values	very high duty cycle	complex data analysis
SLIM	13 m	Q-TOF	MOBILion’s HRIM-MS	∼230–315	requires calibration from known CCS values	resolves peaks separated by as little as ∼0.6% can be installed to multiple MS setups, compact size	requires calibration, not proven for large complexes
FAIMS	few square centimeter area devices	Orbitrap	Thermo FAIMS Pro	<30	not readily achievable	do not pulse ions; acquisition of continuous mobility data without loss of duty cycle	no readily CCS information

a The resolving power, manufacturing name, advantages and disadvantages of the IM platforms are disclosed.^11,171,185,194,199,204,207,208^

### 3.7 Predicting the CCS of Proteins

One of the most compelling aspects of measuring CCS values experimentally is the opportunity to compare such values with those derived from candidate geometries which may be from crystal structure or nuclear magnetic resonance (NMR) structure coordinates, or even a small-angle X-ray scattering (SAXS) measurement. Such comparisons allow the researcher to gain better insights into the conformational landscape adopted.^209−211^ These methods have been well-reviewed^86,99,212−214^ and broadly fall into three categories. In the first category are those that fully evaluate the trajectory of the ion as it interacts with the buffer gas so call trajectories methods (TM) including (MOBCAL-TM and IMoS).^5,93,97,212,215−218^ The second category includes those that consider the projected area of the candidate structure and use empirical data to determine a CCS (PA, PSA, IMPACT).^91,92,95,96,219^ The last category considers the recently emergent machine learning approaches.^12,220−225^ The first two approaches rely on a reasonable starting structure, and commonly with proteins, molecular dynamics methods, both atomistic and coarse-grained that can be used to provide candidate gas-phase geometries. Such molecular dynamics (MD) evaluation can be computationally very expensive, although refinements to this have been made that integrate CCS values into the conformational searching for suitable candidate geometries. We focus here on a couple of examples where MD modeling coupled with IM-MS measurements have been used to examine desolvation. Given the advent of Alphafold^226^ with its ability to predict the crystal structure of a given protein sequence, we in turn predict a refocus in structural biology to measure the conformational dynamics of proteins and other biological molecules both experimentally and computationally, and we believe that IM-MS has an important role to play here. There are remaining challenges in assigning charge correctly and navigating the transition of structure from solvated to dehydrated with force fields that are now primarily only developed for solvated states.^180,210,211^

#### 3.7.1 Comparison of Experimental CCS Values with CCS from MD in Vacuo for IgG Proteins

**Figure 3:**
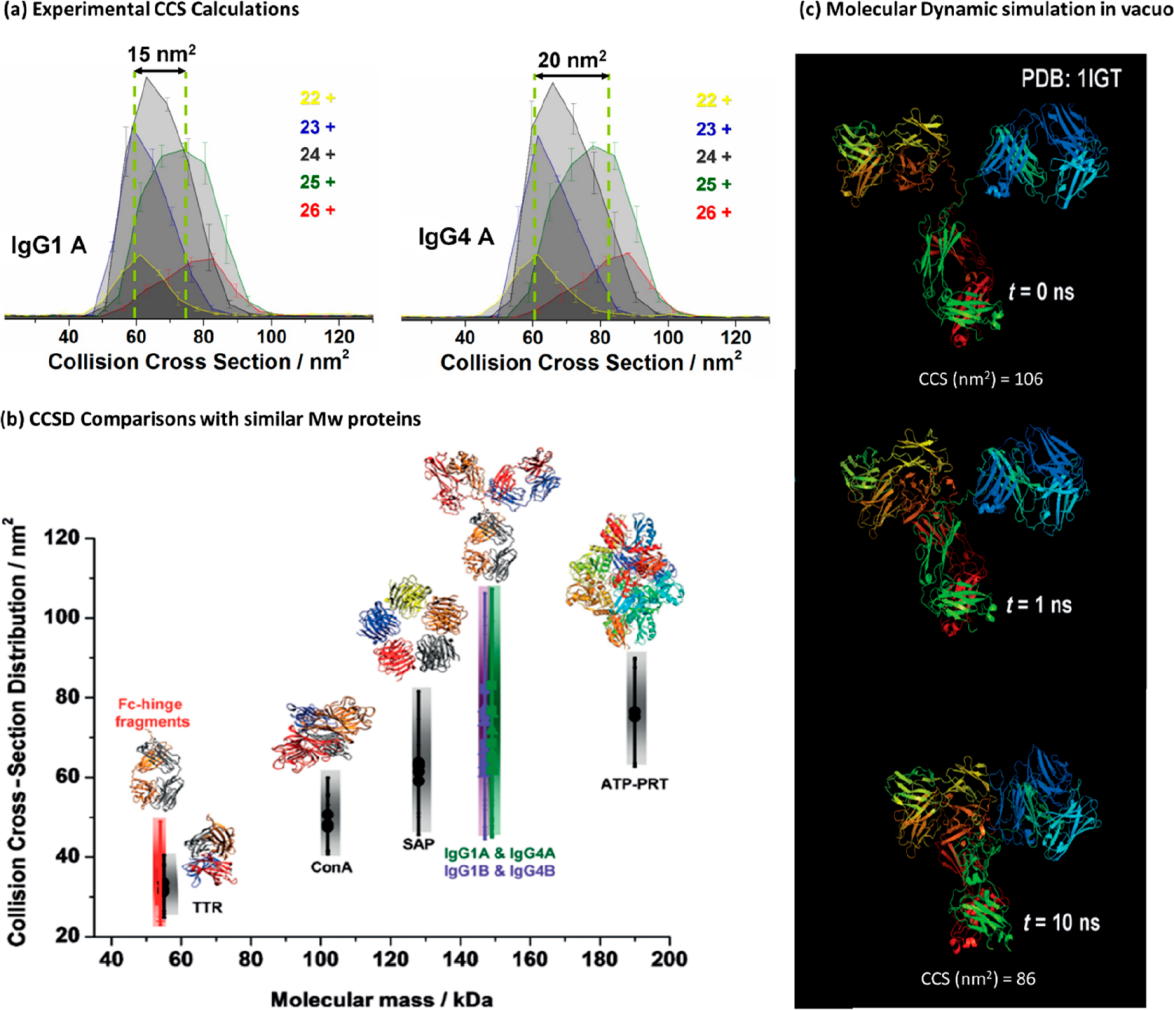
A combination of IM-MS and molecular dynamics (MD) to study the structural differences between two IgG1, two IgG2, and two IgG4 monoclonal antibodies (mAb), of different antigen specificity (A,B). The three classes of mAbs structurally differ in the disulfide bonds’ arrangement around the hinge and Fab domain. (a) The conformational space difference of IgG1A and IgG4A is observed in their median collision cross-sections (CCS), as a 5 nm^2^ difference between the 24+ charge states. (b) The larger range of the collision cross section (CCS) distributions occupied by IgG1A, 1B, 4A, and 4B compared to other proteins of similar molecular weight is indicative of their flexible structure. (c) Comparison between the experimental and MD CCS values unanimously indicated the compaction of the IgG2A antibody, mainly around the hinge region in both IgG1 (not shown) and IgG2. The experimental CCS values are significantly lower than the MD values suggesting structural collapse. Adapted with permission from ref (211). Copyright 2014 Wiley-VCH.

An early example of combining MD coupled with CCS elucidation to support experimental CCS measurements were performed on monoclonal antibodies by Pacholarz et al. (Figure 3).^211^ The CCS distributions for two monoclonal antibody (mAb) subclasses, IgG1 and IgG4, were determined experimentally using DT IM-MS. All mAbs showed broad CCS_He_ distributions ranging from 45 to 85 nm^2^ centered at 66 nm^2^ (Figure 3a). For the MD in vacuo, available crystal structure coordinates for IgGs (IgG1–1IGY and IgG2–1IGT)^227−229^ were used as a basis from which to compare to experimental measurements. At the time these represented the closest candidates for which crystallographic information was in the public domain. The starting coordinates were heated at 300 K and run for 10 ns in total (Figure 3c). Then the CCS values of each mAb across the MD trajectory were calculated using the trajectory method (TM). There was a significant discrepancy (>20%) between the CCS values from the crystallographic starting structures even after 10 ns of MD and the experimental gas phase CCS values. The discrepancy was attributed to structural collapse in the gas phase, which was evident in the dynamics simulation and had been previously highlighted for several proteins.^184,214,230^ The CCS obtained for the IgG2 mAb (1IGT) at *t* = 0 ns, was 106 nm^2^ and by the end of the 10 ns MD had contacted by more than 10% to 86.5 nm^2^. Compaction was also evidence for the IgG1 mAb (1IGY) where the CCS following dynamics was found to be 84.1 nm^2^. The majority of the contraction was found to be in the hinge region (Figure 3c), with much of the secondary structure remaining as it was in the starting coordinates.

Comparing the CCS distributions of the two mAb subclasses to various other proteins of similar molecular weight (Figure 3b) revealed that although the magnitude of the CCS increases proportionally with the mass of the proteins, the CCS distributions of the IgGs are significantly broader, indicative of higher flexibility and more conformers.

#### 3.7.2 Molecular Dynamics Workflow for High Accuracy Predictions of IgG CCS values in vacuo

**Figure 4:**
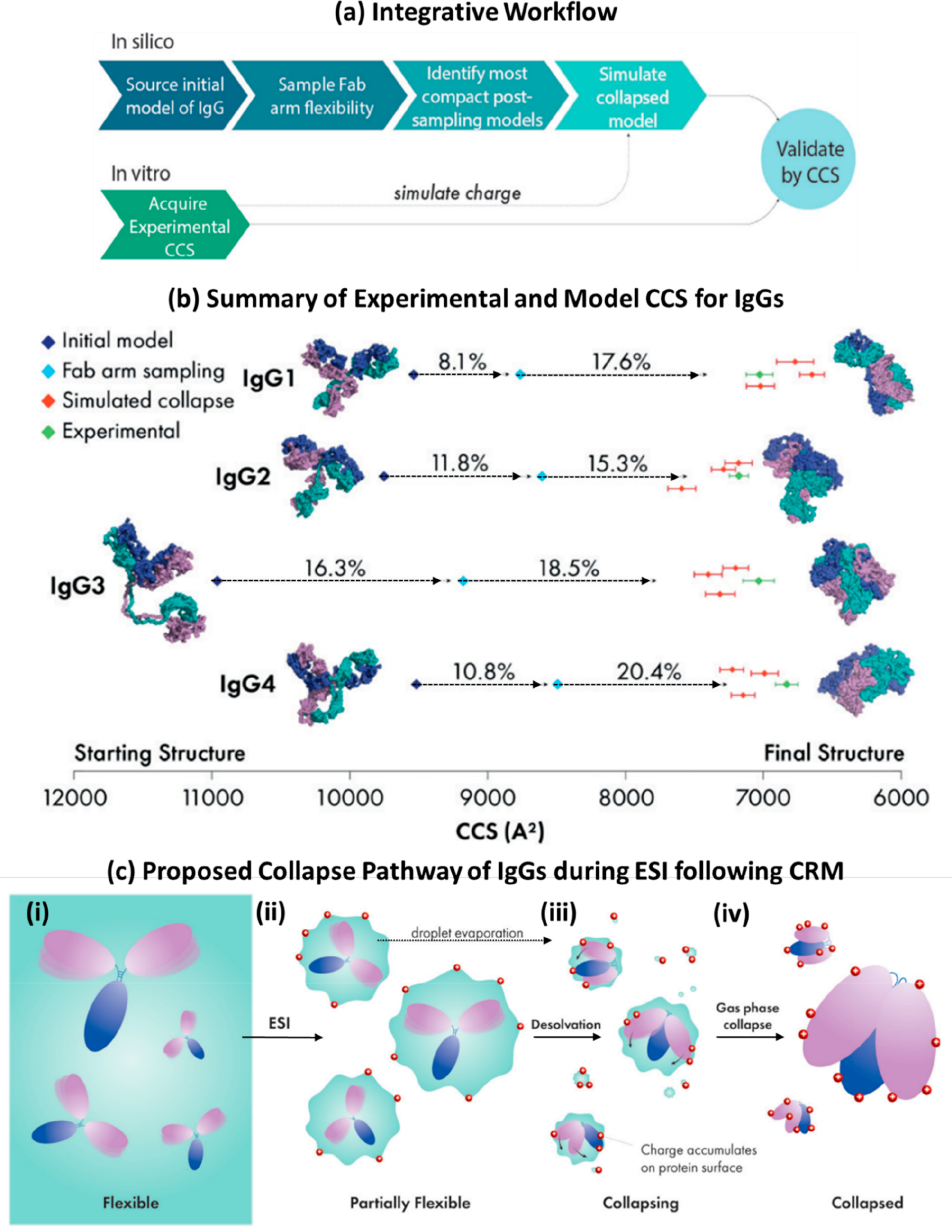
A combination of experimental data and molecular dynamics (MD) simulations to describe the contraction pathway for all four subclasses of antibodies (IgGs) during electrospray ionization (ESI). (a) The integrative workflow samples compact IgGs following MD, using IMPACT calculated CCS values and then progress these structures for further minimization, it also reports on the IgG flexibility. (b) The collision cross section (CCS) values were calculated at each stage of the simulation, and the CCS reduction found is shown in percentage terms. The MD CCS values were then compared against the experimental data (green dots). The green error bars represent the standard deviation of the readings. The simulated collapse models (red bars) were run in triplicates, and the red error bar indicates the CCS range over the last nanosecond of the simulation. (c) The proposed contraction pathway is shown in steps i–iv, which also follow the charge residue model (CRM). (i) The antibody is flexible in solution. (ii) Sample undergoes electrospray ionization (ESI). (iii) Solvent droplets undergo evaporation. (iv) The structure progressively undergoes compaction mainly on the Fab domain and the antibody is now desolvated in the gas phase. The red spheres indicate the charge. Adapted with permission from ref (231). Copyright 2018 Wiley-VCH.

Politis et al. extended this work with a more advanced MD workflow to simulate more accurately the desolvation process of ESI, as well as consider the subdomain movement more explicitly.^231^ They integrated the calculated CCS values from the MD trajectories (1) initially before any desolvation, (2) postsampling, and (3) gas-phase with those found experimentally (Figure 4a).^231^ The calculated CCS values were generated by IMPACT using a scaled projection approximation (PA) method.^92,99,230^ The experimental CCS values were used to train the selection of structures from the MD trajectory to find candidate geometries that would best represent those observed. The difference in CCS of ∼30% between initial structures and experiment was also indicative of substantial contraction in agreement with the prior findings of Pacholarz et al.^211^ Following the iterative refinement process, candidate structures for the gas phase conformers were found where the agreement in CCS was 6% for IgG1, IgG2, and IgG4 and 0.1% for IgG3. The proposed contraction during ESI is shown in Figure 4b,c, where the mAbs gradually progress to a more compact structure while desolvation takes place. These finding are in line with work performed by Konermann and co-workers on monomeric proteins (ubiquitin, cytochrome c, and holo myoglobin)^7^ and by Beveridge et al. in studies on charge segregated disordered proteins^180^ each using MD trajectories >100 ns in length. Despite increasing timescales and better agreement with experiment, none of these extensive MD studies performed to date come close to properly represent the ∼ms time scale of desolvation in IM-MS experiments.^128^

#### 3.7.3 Integration of Native IM-MS with Molecular Dynamics to Determine the Architecture of Heteromeric Protein Assemblies

With the structural complexity and conformational heterogeneity of protein complexes, it is often challenging to obtain crystals for structural determination with classical high-resolution structural biology tools such as X-ray crystallography and NMR. Often, it is found that domains of the proteins (N- and C-termini) need to be truncated to aid the crystallization process resulting in their absence from the crystal structures found in the PDB.^232−234^ Because mass spectrometry does not “care” if a given protein can crystallize or not, it is highly suited to the study of WT forms of proteins in any given complex, although as discussed above it is less forgiving of mass heterogeneity. This then raises the challenge of how to manipulate existing structural data into a form that can be compared with experiment. This challenge has been met by many groups in different ways^149,184,195,235−245^ and as a pertinent example of to deal with incomplete crystal structures to allow comparison with IM-MS data, we here refer to work from Politis et al., who integrated IM-MS experiments with MD simulations to elucidate structures of heteromeric protein assemblies.^245^

In this study, the authors developed an algorithm to construct higher-order oligomers trained on packing arrangements that best agree with the IM-MS experimental data. They tested two possible starting points to this approach, in the first a high-resolution structure is available from other methods which is used to form an initial building block that can be compared with IM-MS CCS values. The second route is via an incomplete structure which is refined using homology models. If such homologous models are not satisfactory/available, novel coarse-grain (CG) approaches can then be deployed. This workflow was successfully applied to numerous protein complexes with different levels of structural data, such as multimeric protein complexes within the *Escherichia* colireplisome: the sliding clamp, (β2), the complex (c3dd9), the DnaBhelicase (DnaB6), and the single-stranded binding protein (SSB4).

**Figure 5:**
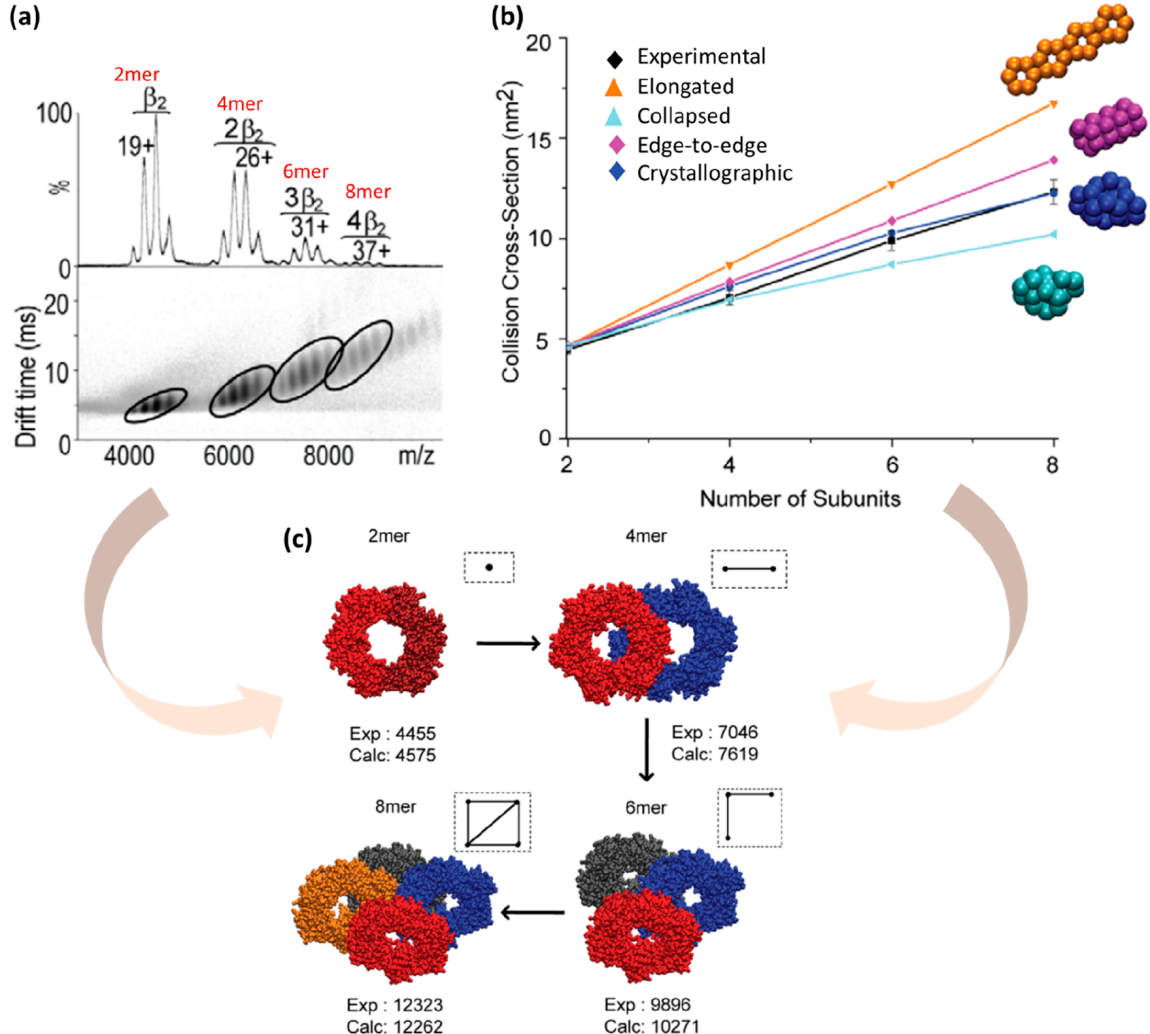
Assembling of atomic structures for the oligomeric species of the sliding clamp specie (β_2_). (a) Mass spectra and native ion mobility mass spectrometry (IM-MS) measurements are acquired for both intact and subunits which were generated in solution. (b) Collision cross section (CCS) values are calculated using MOBCAL Projection Approximation for different model low resolution structures and plotted against the different numbers of subunits. The theoretically calculated CCS are compared against the experimental CCS (black) to satisfy the IM constraints of 7%. The CCS from the crystallographic structure (blue) and the experimental CCS have high agreement. Similarly, the edge-to-edge structure (purple) has good agreement with the experimental CCS until the 6mer level, after which it starts deviating. Upper and lower limit of the CCS are represented as an elongated (orange) and collapsed (cyan) structure. (c) To build the high-resolution oligomers, the building block was used. Structural information to build such topologies were mined form the crystal symmetries with the best fit from (b). Atomic models for the oligomers were constructed once the calculated CCS from the atomic model were in good agreement with the calculated CCS. The inset provides a representation of the topological arrangements of each oligomer. The dots represent the center of mass of each dimer and the edges the interconnections. Reproduced with permission from ref (245). Copyright 2010 Politis et al., published under the CC BY 4.0 License http://creativecommons.org/licenses/by/4.0/

In this section, we will discuss their findings for the sliding clamp complex (β2) application, whose complete structure is available in the PDB.^246,247^ Using both native MS and native IM-MS, the heterogeneity of the sample (2mer, 4mer, 6mer, 8mer) along with their CCS values were measured and the latter compared across the theoretical CCS values calculated using the Projection Approximation (PA) in MOBCAL^218^ with close agreement (Figure 5a,c).The building block is used to generate various structural arrangements (Figure 5b) and their CCS values are again estimated using one of the PA based algorithms. The highest and lowest CCS values are used as the upper and lower limits for the topology search (Figure 5b, orange). For the whole range of subunits, very close agreement (within 7% error) in CCS was observed between the experimental (black) and crystallographic (blue) measurements, which proved to be the most-likely architecture for the 2- to 8-meric structures (Figure 5b). Similarly, the end-to-end structure indicated high agreement until the 6mer (purple) level, after which there is significant deviation. Using both the IM-MS data and the most-likely architecture findings, atomic model structures are developed and accepted once they are of good match (within 4%) of the experimental CCS values for the higher oligomeric complexes. The possible structural packing with the most confidents is the compact (blue) (Figure 5b) topology mined from the crystallographic structure of the end-to-end topology between dimers.

#### 3.7.4 Simple Toy-Model to Approximate the Range of CCS a Protein Molecule Can Hold Requiring Only the Sequence as an Input

**Figure 6:**
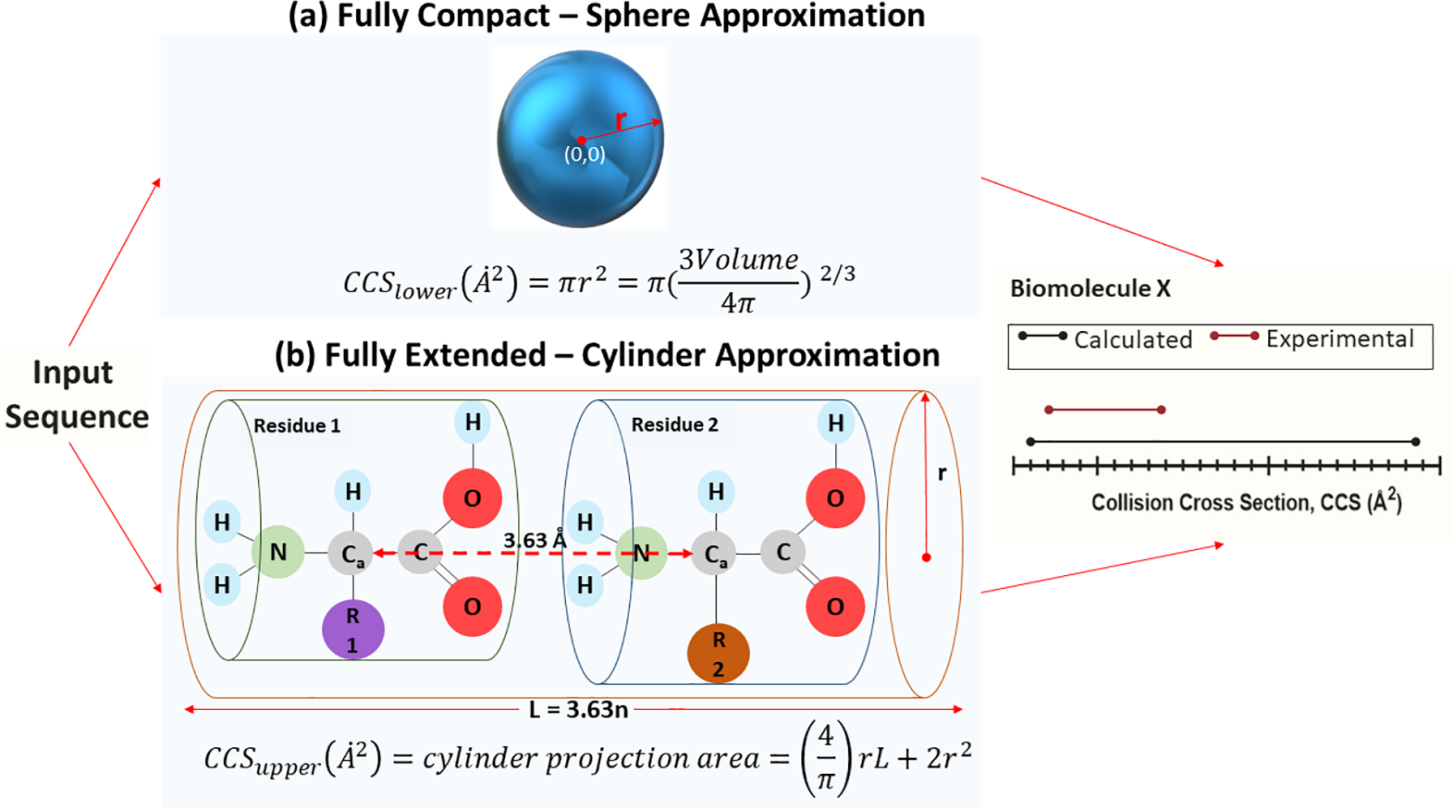
Schematic of the toy model first introduced by Beveridge et al.^145^ The lower and upper limit for the collision cross section (CCS) range a biomolecule can hold can be calculated using only the sequence as an input. The lower limit is calculated by approximation of the sequence to a sphere (a), whereas for the upper limit the sequence is approximated to a cylinder with a maximum distance between the alpha carbons of 3.63Å (b). For biomolecules with various domains, the longer chain is used for the toy-model calculations.

To provide a simple framework to consider the range of CCS values that could be adopted by any given protein, in a globular and a fully extended state we developed a toy model.^145,146^ This method has particular relevance to highly dynamic and disordered proteins. It uses a simple approximation where fully folded biomolecules are assumed to be spherical, with a density determined by the mass and the average density of proteins in the Protein Data Bank (PDB), and the fully extended form is represented by a cylinder with a length (*L*) that is equivalent to *N* × 3.63 Å, where *N* is the number of amino acids in the protein and 3.63 is the distance between alpha carbons (C_a_) and the radius (*r*) of the cylinder is found from a weighted average of the size of the R groups of each amino acid (Figure 6).

This model was trained on both structured and disordered proteins, namely α-synuclein, β-casein, myoglobin, lysozyme, and cytochrome c. This predicted range is compared with the experimental range of CCS values found for each protein (Figure 6). This can be used to determine if the protein is primarily presenting globular forms (e.g., natively folded) or is more conformationally dynamic (e.g., disordered).^148,180,248^ This toy model has proved to be instructive in the study of protein conformation but certainly has limitations and ought not to be used to replace experiment nor prevent more extensive MD based conformational refinements to find candidate geometries for comparison to experiment.

##### 3.7.4.1 Applications of Toy-Model and MD to Examine an IDP and Its Complexes

###### 3.7.4.1.1 Intrinsically Disordered Proteins (IDP): Cell-Cycle Regulator, p27

Extensive MD simulations such as those described in section 3.7.2 as well as the toy model described in section 3.7.4 were both used in studies of the cell-cycle regulator protein, p27.^148,180^ These give some indication as to where MD is possible and where the toy model is useful. Full-length p27 (p27-FL) is a well-known intrinsically disordered protein (IDP) and is composed of two distinctive domains: (1) p27-KID is the N-terminal kinase-inhibitory domain and (2) p27-C is the disordered C-terminal domain. Extensive MD to mimic the desolvation process was applied to find suitable structures of the highly disordered C terminus. In this work, converged solution-phase Metropolis Monte Carlo (MC) simulations reported by Das et al.^249^ were searched to find compact forms of the protein which were selected for subsequent MD. These compact starting structures of p27 were first solvated in ∼6000 water molecules which were then removed sequentially to leave the desolvated protein. In order to account for the possible location of protons on the protein a total of 5000 protomers were constructed. These protomers were iteratively segregated and selected based on their charge state and taken on further based on lowest energies following minimization and equilibration. As a consequence of this extensive atomistic MD approach, a conformational ensemble that better represented experiment was found.

**Figure 7:**
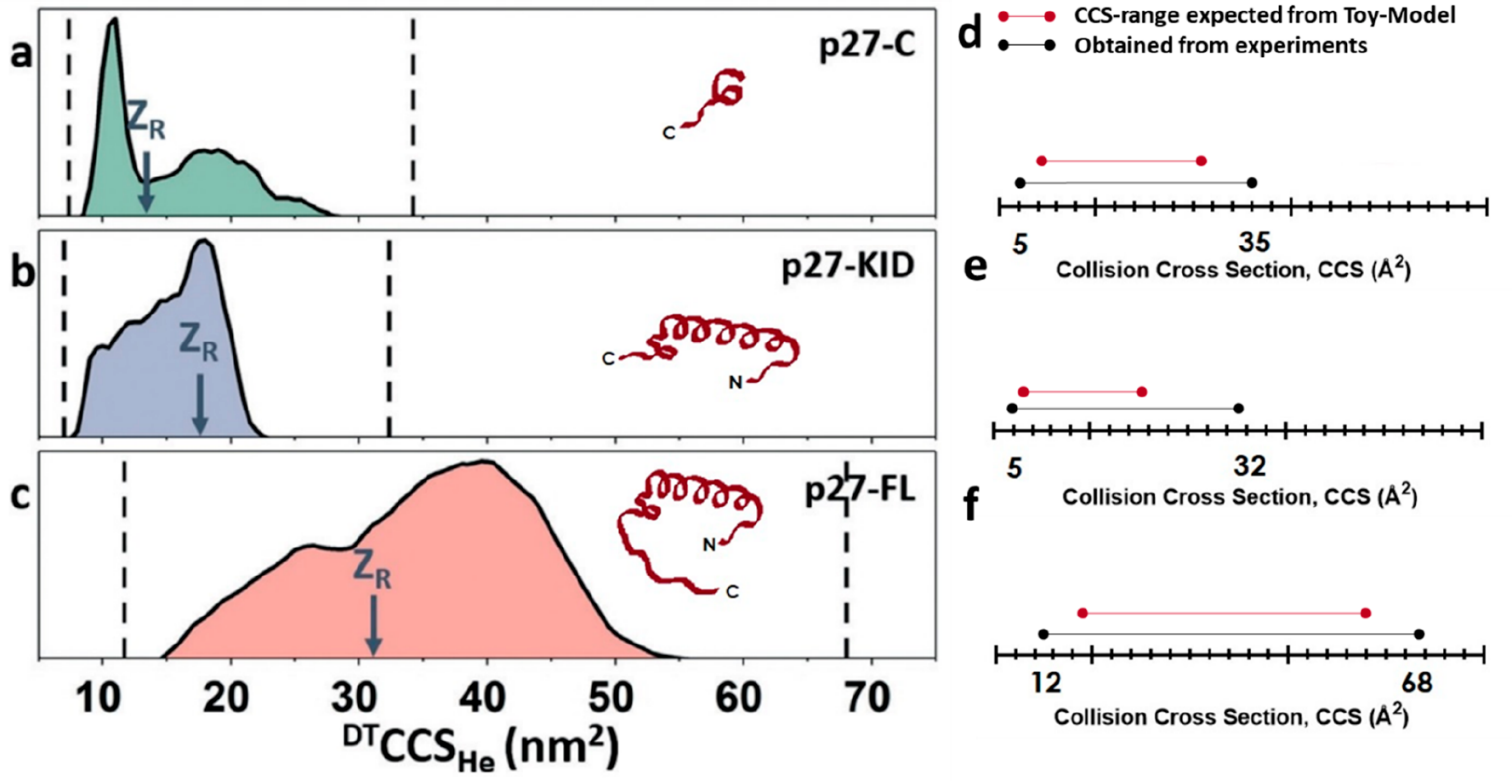
Global collision cross section (CCS) values obtained for the disordered C-terminal domain (p27-C) (a), the N-terminal kinase-inhibitory domain (p27-KID) occupying ∼50% of the theoretical CCS range calculated by the toy-model (b), and full-length p27 (p27-FL) occupying ∼75% of the theoretical CCS range calculated using the toy-model (c). The flexibility of p-27 seems to be imparted by the c-terminus. The vertical black dash lines represent the lower and upper limit or the CCS range p27-C (d), p27-KID (e), and p27-FL (f) could hold, as calculated by the toy model above. On the global CCS plot, the Rayleigh limits (*Z*_R_) are also annotated by blue arrows. Adapted with permission from ref (148). Copyright 2019 Wiley-VCH.

Such simulations were possible for the 11.2 kDa C-terminus of p27, but this would have been computationally prohibitive for the intact protein. Experiment was, of course, well able to examine the full-length protein and conformational distributions can be compared to the predicted extremes of CCS values that the full-length p27 (Figure 7c) and each of the two domains could occupy (Figure 7a,b). By using the toy model, it is trivial to see that the full-length protein and the C terminal domain alone are both able to occupy 75% of the predicted possible CCS range (Figure 7a), whereas the N-terminal KID domain is more compact and only occupies 50% (Figure 7b), implying that most, but not all, of the conformational flexibility of this protein is imparted by the C terminus. Further work showed how this flexible protein is tamed when complexed to Cyclin:CDK.

To allow the reader to use this toy model, we have included a spreadsheet and instructions in our supplementary data.

## 4 Advanced IM-MS Methods and Instrumentation

**Figure 8:**
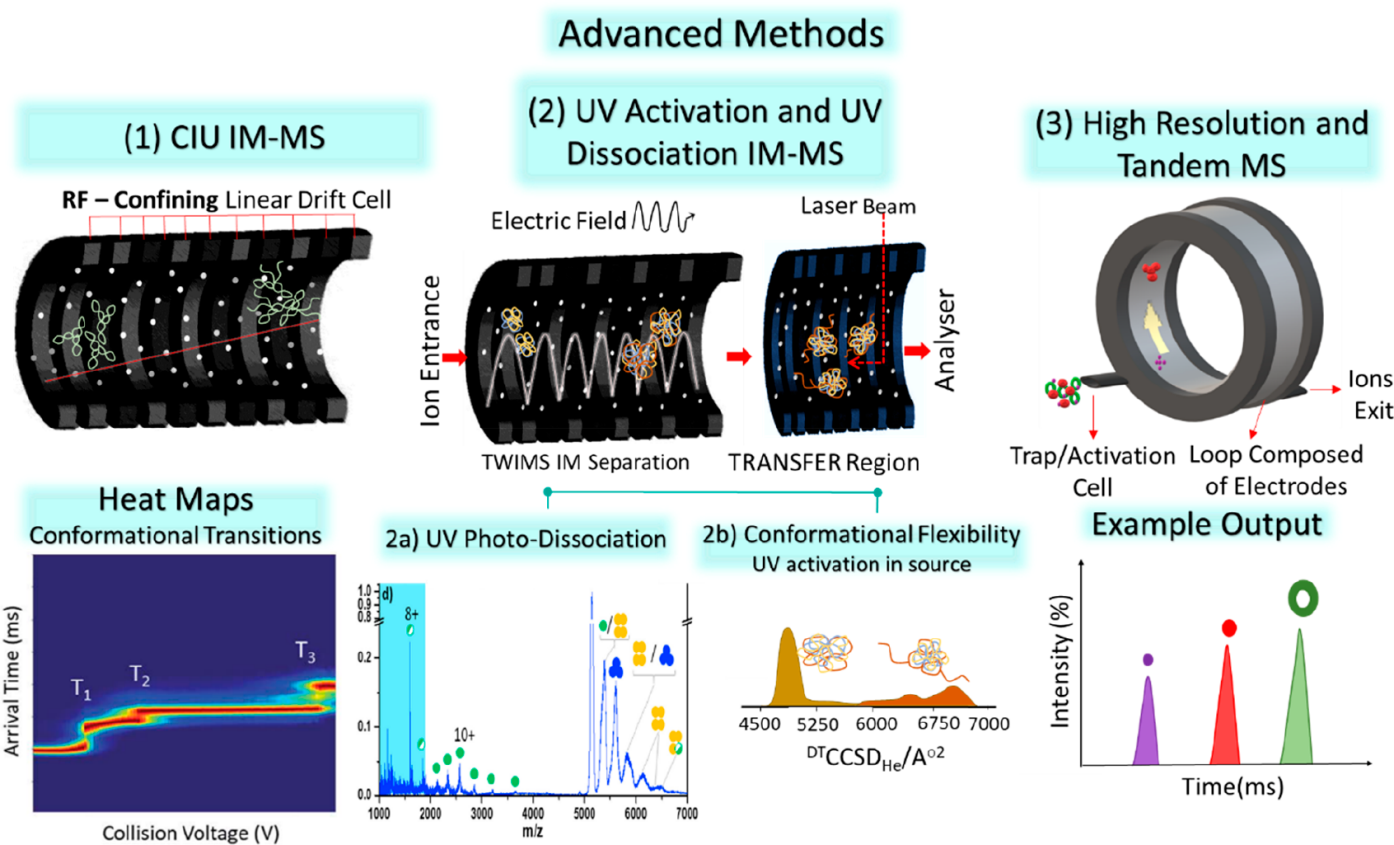
Advanced ion mobility mass spectrometry (IM-MS) methods and instrumentation. (1) During collision-induced unfolding (CIU) or activated IM-MS analytes are activated with the use of a voltage ramp. The unfolding patterns and hence structural stability are investigated with the use of the generated heat-maps. (2) During ultraviolet (UV) photodissociation and/or UV photoactivation IM-MS, a laser beam interacts with the trapped analytes pre- or post-IM separation. It has been shown that dissociation can be improved by further applying voltage in the transfer region. (2a) A UV dissociation example where the 20+ tetramer ConA fragments into its smaller subunits (monomer, green; trimer, blue; tetramer, yellow dots). (2b) A cartoon of UV-activated proteins being separated using IM-MS. (3) Cyclic IM-MS enables multiple passes through an approximately 1 m electrode loop, enhancing the ion separation and resolution. Heat map and UV photodissociation graph were reproduced with permissions from refs (256) and (257). Copyright 2019 Royal Society of Chemistry and 2021 Elsevier BV, respectively.

Within IM-MS apparatus as well as measuring the mass and conformation of a given protein complex, it is possible to perform experiments that probe the stability of a given conformation (Figure 8). Mass spectrometry methods that probe protein stability is a topic has been extensively reviewed recently,^250^ and we describe here a few pertinent examples where IM is central to the experiment. Protein ions can be activated in different regions of the instrument, including the source, before and after IM measurements. In all time dispersive IM experiments, ions are trapped and released into the drift cell, so activation prior to this can be used to examine restructuring and unfolding. For Synapt and Cyclic instruments, it is possible to do this following mass selection, whereas for other configurations, for example, the Agilent 6560 series and the Waters Vion, the IM cell is located before the mass selective quadrupole. For activation post IM separation, the effect of activation on the structure will not be reflected in the IM data. Such experiments can be used to obtain sequence or subunit information from different conformers because the products can be tracked to the precursor ion ATD. Depending on the aim of the experiment, activation in any of these regions may be performed to further desolvate, induce subunit or covalent bond dissociation, and for perturbation of the fold. Numerous well-characterized methods may be used to activate the gas phase protein ion, including (1) collisional induced dissociation (CID),^251^ (2) electron transfer dissociation (ETD/ECD),^244^ surface induced dissociation (SID),^252,253^ and (3) ultraviolet photo-dissociation (UVPD).^254,255^ We discuss below how each dissociation method can be used in an IM-MS experimental workflow to obtain rich structural data for biological macromolecules.

More advanced versions of the simpler IM-MS instrumentation described in section 3 have been developed, including instruments with very long drift tubes and even cyclical IM geometries aimed at increasing the resolution of the measurement and enabling tandem IMS experiments.^164−166^ Both long drift tube and cyclical geometries have been commercialized. In 2016, Agilent developed the 6560 IM Q-ToF,^171^ with a 1 m long drift tube, and in 2019 following the pioneering first cyclical geometry developed by Clemmer et al.,^164^ which relied on a somewhat convoluted arrangement of DTIMS and ion funnels. Waters developed a commercial version that makes use of a TWIMS geometry that forms an electrode loop (∼1 m circumference).^165^ Such geometry provides the opportunity for enhanced ion separation, by increasing the drift tube length and hence the resolution. Analytes can be directed around the electrode loop multiple times, to separate isobaric species that have a very small difference in their mobility, reaching resolutions in excess of 300.

### 4.1 Collision-Induced Dissociation (CID)

Low energy CID conditions (1–200 eV),^258,259^ are commonly employed to probe protein restructuring. In such, analytes are subject to ∼10^3^ to 10^5^ gas collisions either in the ion guides before or after the IM analysis. In most instruments, to achieve higher energy collisions (2–10 keV),^258^ ions are accelerated through the collision gas by increasing their initial voltage. Many studies have sought to confirm stoichiometry and determine the topology of protein complexes following native MS with CID.^49,238,260,261^ These cannot often provide structural insight into the precursor form of the homomeric complexes. The main reason is that the time scale for CID activation is micro- to milliseconds,^262,263^ which allows for substantial structural rearrangement before the ejection of a single highly charged monomer and the remaining part of the complex with lower charges.^49,259,264^ The structural rearrangement before dissociation is the result of the extensive low-energy collisions that slowly increase the internal energy of the ions over time. The dissociation is nonspecific and usually occurs at the weakest bonds. High-energy CID, which involves activation energies up to the keV scale occurs over a few μs, which decreases the opportunity for structural rearrangement. Many studies have combined IM-MS and CID to provide information on the disassembly of protein complexes,^49,174,260,260,261,265^ and it is commonly asserted that the fragment ion structures and stoichiometries have arisen from rearranged intermediates that differ from the native assembled form.

#### 4.1.1 Use of IM-MS Combined with CID to Probe Protein Disassembly

##### 4.1.1.1 Transthyretin

**Figure 9:**
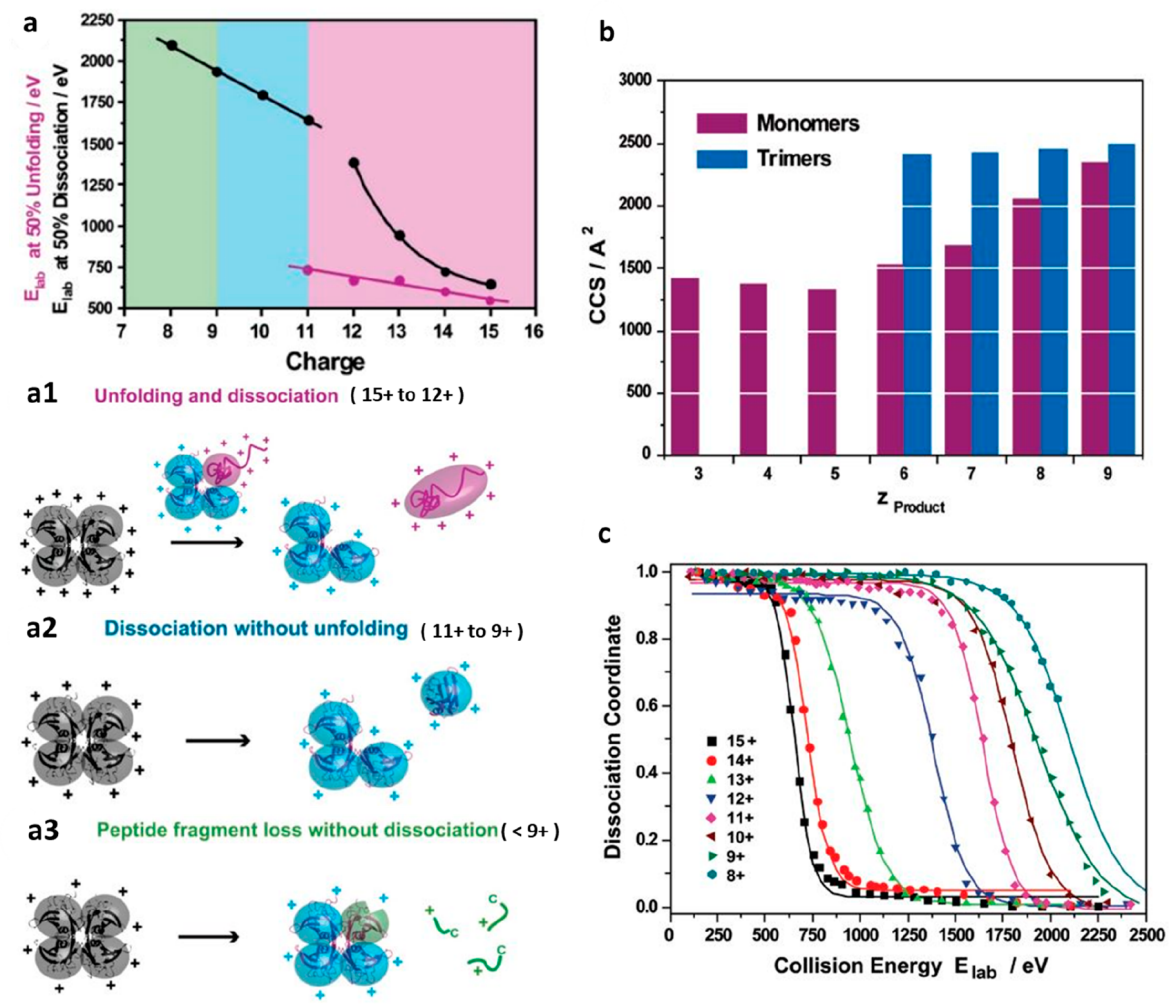
Use of collision induced dissociation ion mobility mass spectrometry (CID IM-MS) to investigate the impact of the homotetrameric transthyretin protein’s (TTR) precursor charge state on the protein disassembly. (a) The initial charge state (precursor) of TTR impacts the energy requirements for unfolding and dissociation. The plot is color coded for the dissociation pathways: (a1) highly charged precursors follow the well-known asymmetric charge-partitioning pathway to release a monomeric product, (a2) intermediately charged precursors dissociate into a monomer and a trimer, and (a3) very lowly charged precursors undergo fragmentation producing C-terminal fragments without prior subunit unfolding or dissociation. (b) CCS of subdomains released at different charge states. Monomers with high charges have higher CCS value (extended) and lowly charged monomers are more compact with lower CCS values. The CCS of the trimers is unaffected by the charge state of the precursor. (c) Dissociation profile in which dissociation coordinates represent the relative amount of intact tetramer present. Lowly charged precursors require higher amounts of collision energy to produce the same dissociation coordinate value as a higher-charged precursor. Adapted with permission from ref (259). Copyright 2010 American Chemical Society.

The assertion that collisional activation will first disrupt the native fold prior to dissociation was confirmed in foundational IM-MS investigations by Ruotolo, Robinson, et al., examining the homotetrameric transthyretin protein, TTR (55 kDa), where the CCS of the departing monomers was very large^259,266,267^ (Figure 9). The homotetrameric TTR that was prepared in ammonium acetate (AmAc) presented in charge states 13–15, triethylammonium acetate (TEAA) 10–12 and with a crown ether (CEs) buffer with charge states below 10+. TEAA is an additive that reduces the charge on the desolvated ion without substantial interference to the conformation of the analyte.^142^ Here the authors investigated how the charge of the precursor impacts the conformation variation of the ejected monomers and trimers post CID (Figure 9). The charge of the product ions highly depends on the initial charge uptake of the precursor. In other words, lowly charged precursors lead to lowly charged product ions which translated to more compact monomer conformations and vice versa (Figure 9a1–a3). In marked contrast, the CCS values of the trimer released from the differently charged precursors did not change (Figure 9b). This observation was validated by the CCS values obtained from their IM-MS experiments on the released monomers and trimers (Figure 9b). One of the key findings is that lowly charged precursors require less energy to unfold and more to dissociate (Figure 9c). As a result, the use of charge-reducing agents has proven to increase the stability of biomolecules in the gas phase by preventing unfolding transitions and requiring high collision energies to undergo dissociation.^259^ Such approaches can have impact where it is important to maintain the native structure of biomolecules and their subunits for mass and mobility measurements.^268,269^

##### 4.1.1.2 UVR8 Plant Photoreceptors

An example of where CID coupled with IM-MS revealed the stability of the gas phase form of a protein complex compared with that found in solution was a study examining the full-length UVR8 photoreceptor.^270^ UVR8 plant photoreceptors absorb UV-B light to regulate protection and photomorphogenic responses in plants. UVR8 is an inactive homodimer in the dark, and when it absorbs UV-B light is converted to an active monomeric state.^271^ The UVR8 molecule consists of a β-propeller core domain and intrinsically disordered N and C-terminal tails. To crystallize this protein, the N and C termini had to be removed, resulting in a stable dimeric form,^270−273^ which was often used as a proxy for the active protein. Here a hybrid IM-MS approach was used to analyze the conformations adopted by the full-length photoreceptor upon light activation.^270,274,275^ Initial experiments tried UVPD to dissociate either the core domain or the wild-type (WT) dimer in the gas-phase. This was unsuccessful and only produced small fragments from the N and C termini. A similar observation was made with CID, which was unable to break up the dimeric interface. This was instructive because the complex was irradiated in the ESI tip, it was trivial to see the ensuing monomer and show the difference between the solvated and the gas phase structures. Despite the inability to dissociate the subunits of this dimer, IM-MS was used to monitor the conformational changes that occur to the UVR8 protein complex following collisional activation which is covered in section 4.2 below.

### 4.2 Collision-Induced Unfolding (CIU): Activated IM-MS (aIM-MS)

A method whereby a given biomolecule is activated by collisions or photons or electrons and the result of this activation is examined using IM as well as MS is accurately described by the term activated ion mobility mass spectrometry, aIM-MS.^276^ The more widely used term CIU refers to the more specific case wherein the experiment is concerned with viewing the collision-induced unfolding of the molecules.^250^ This term is not fully descriptive of the processes that occur upon activation. Even in the case of protein complexes both compaction and dissociation can also occur. CIU is best considered as one output following activated aIM-MS.

**Figure 10:**
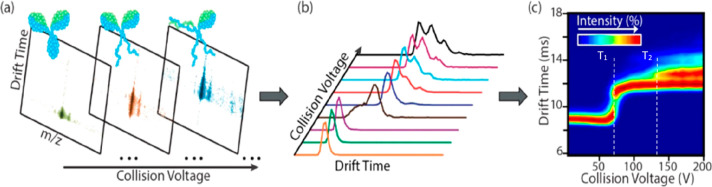
Collision-induced unfolding ion mobility mass spectrometry (CIU IM-MS) workflow using a monoclonal antibody (mAb) as the primary analyte. (a) An isolated ion of a specific charge state undergoes collisional heating by increasing the collisional voltage of the trap ion guide before the IMS cell. Increase in collision voltage results in structural unfolding and consequently increase of drift times. Dots indicate more spectrums acquired for intermediate collision voltages. (b) Drift times are recorded for each collision voltage applied during step (a). (c) A CIU contour plot (heat map) is produced, where the transition stages (unfolding) of the mAb are denoted by T_1_ and T_2_ . Adapted with permission from ref (284). Copyright 2015 American Chemical Society.

Most commonly, biomolecules are activated before the IM cell, but other activation areas can also be used, such as between the spray tip and the entrance to the mass spectrometer or within the desolvation ion optics. In mass selective IM-MS instruments, a single charge state ion can be isolated and exposed to a collisional voltage ramp which for unmodified Synapts typically ranges between 5 and 200 V, (Figure 10). We have made software available that allows such ramps to be customized from linear increase to, for example, an exponential function.^276^ Recently, Vallejo et al. modified the Agilent 6560 by including an additional lens (termed a fragmentor) at the end of the ion transfer capillary and managed to increase this collisional voltage range up to 560 V.^172^ Increasing the collision voltage results in more energetic collisions between the analyte and the gas, which will eventually promote restructuring (Figure 10a). By recording the ATD at each collisional voltage, a heat map showing the change in the mobility of the ion with respect to collision energy can be produced, typically this shows an increase in CCS as the voltage is ramped and also reveals the unfolding intermediates often with exquisite detail (Figure 10b). Such heat maps act as fingerprints that provide the structural stability of a given biomolecule for example as a function of ligand binding,^266,277−281^ mutations,^282^ polydispersity^283^ or to compare proteoforms (Figure 10c).^256,284^ There are numerous softwares available to analyze CIU data, such as: CIUSuite^285^ and Origami.^276^ For further details on applications of CIU and on the available software for analysis and interpretation of IM-MS data, we direct the reader to two excellent recent reviews,^99,250^ and here we highlight some examples that exemplify the use of the method.

#### 4.2.1 Classical Biophysical Techniques Used to Probe the Stability of Proteins

A range of reference techniques are daily deployed to characterize the structure or conformational state of proteins in order to measure their stability,^286−297^ namely, differential scanning calorimetry (DSC),^298−305^ differential scanning fluorimetry (DSF),^306,307^ circular dichroism (CD),^308,309^ and isothermal titration calorimetry (ITC),^310,311^ and analytical ultracentrifugation (AUC).^312,313^ These methods, which are often employed in concert with IM-MS data, also provide global information on the structure and stability of proteins, however, they all provide averaged values on the conformational ensembles because the readouts are the summed signal output from various parts of the structure probed, compared with gas phase methods which probe isolated biomolecules and hence conformational populations. As an example, CD readings provide the average percentage of the basic types of secondary structure (α-helixes, β-sheets, coils) present within the analyzed sample. In samples with a lowly populated conformational state minimal differences that this adds to the bulk measurement may not be visible to the analyst but may be captured by MS methods, both protein centric as discussed in section 4.2.2 as well as peptide centric such as hydrogen–deuterium exchange mass spectrometry (HDX-MS).^314−322^ In addition, bulk biophysical methods are often unable to provide insights regarding the effects of any given modification to the biomolecule structure. By contrast MS methods, both protein and peptide centric, require small volumes of sample, they are highly sensitive to structural changes, have short acquisition times, can isolate modified (mutated, oxidation, acetylation, methylation, PTM) regions on the sequence, and can easily relate such regions to the stability, dynamicity, or even aggregation propensity^316,323−330^ of proteins.

#### 4.2.2 Use of CIU/aIM-MS to Probe the Structure of mAbs for Therapeutic Use

The huge increase over recent years in the development of mAbs and other biological products as therapeutics has provided significant challenges for their analytical characterization. They are of high molecular weight, are inherently polydisperse due to post translational modifications, and can present batch to batch variation, and often are prone to aggregation at the therapeutic dose level.^331−335^ A great advantage of native CIU IM-MS experiments is that they are both quick and highly sensitive to small structural changes that can lead to small stability shifts.^335^ Consequently, this method is highly suitable to examine the structures and stabilities of biological therapeutics.^256,284,326,336−338^ In a pioneering study, Tian et al. used CIU IM-MS to examine mAb–biotin conjugates, and while native IM-MS with no activation step could not differentiate between the small conjugates, CIU experiments could.^284,339^ Similar benefits were reported by Hernandez-Alba et al., who were able to distinguish closely related IgG4 mAbs species by generating CIU IM-MS heatmaps.^340^ These act as fingerprints and provide structural signatures for the IgG4 subfamily, which included a wild-type, a hinge-stabilized (hs), a IgG4 mab with a S228P mutation, and a bispecific IgG4 mAb (bsAb). While naturally occurring mAbs are only specific to a single antigen/epitope, bispecific mAbs are designed to bind to two different antigens or two different epitopes on a single antigen and are a particularly attractive class of therapeutic. However, they often only differ in mass from monospecific mAbs by 1–2% and are even difficult to distinguish with LC-MS methods. Hernandez-Alba et al. produced CIU fingerprints, which indicated that the S228P mutation stabilizes the gas phase conformation and also that bsAb has structural memory of its origin, which is two-parent wt-IgG4s.^340^ Desligniere et al. performed CIU on a higher resolution cyclic IM-MS instrument differentiate between isoforms of trispecific mAbs, showing that improvements in IM resolution can assist in resolving CIU fingerprints,.^341^ This allowed them to distinguish between two isomeric forms of a trispecific antibody (tsAb) by comparing their gas-phase stability. Huang et al. identified how three different epitope locations affect the structural stability of given antigen–antibody complex.^342^ Using fingerprint maps, they were able to isolate one out of the three antigen–antibody complexes that had different binding topology and stability and proved that CIU can be used to classify antigen–antibody complexes based on their epitope maps.^340^

Similar to the work cited above,^337^ Yuwein et al. also used CIU to study a model antibody–biotin conjugate complex and revealed some structural destabilization upon biotin conjugation.^337^ Watanabe et al. employed CIU to show how domain exchange of an anti-HIV IgG1 mAb is shut down by an engineered mutant.^343^ Finally, CIU has been used by many groups to correlate glycosylation and disulfide bonding patterns to the stability of antibody biotherapeutics. Some further examples are discussed in more detail in the following sections.

##### 4.2.2.1 Glycosylation Variation Impact on Conformational Stability, Spread, and Population of mAbs

**Figure 11:**
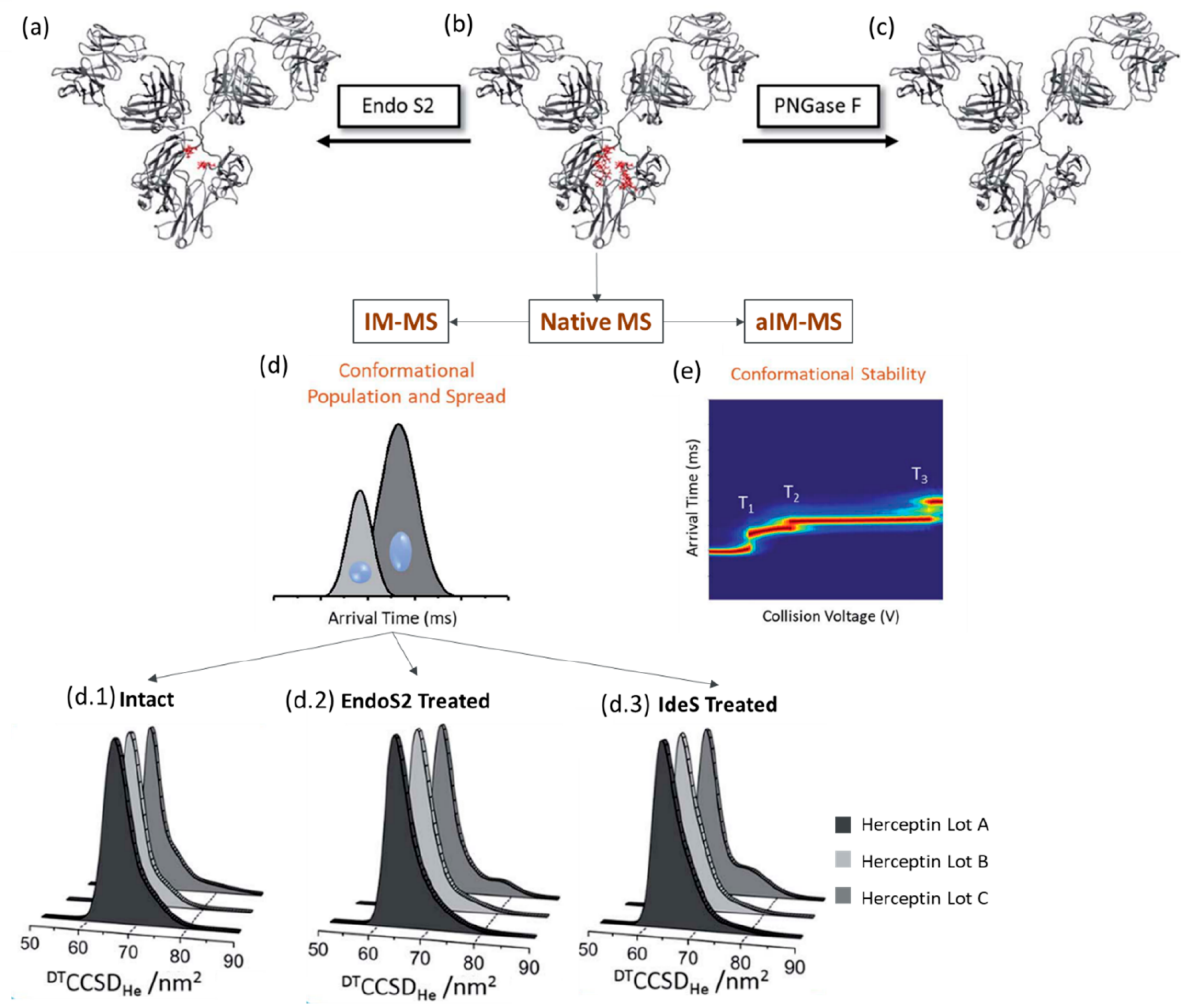
Workflow used to identify the glycosylation impact on the structural stability, conformational populations, and of the therapeutic monoclonal antibody (mAb) herceptin, for three different lots. Each lot was treated (a) with EndoS2, which cleaves between the two innermost GlcNAc residues of the mAb glycans, (b) nontreated (intact), and (c) with PNGase, which cleaved off the mAb glycans between the innermost GlcNAc and asparagine residues. (d) Ion mobility mass spectrometry (IM-MS) revealed similar distributions of collision cross-section distributions (CCS) for lot A and B across the various glycosylation stages (d.1–d.3) and a comparatively broader CCS distribution for lot C. (e) Activated IM-MS (aIM-MS) indicates decreasing stability with decreasing glycan length; intact < deglycosylated (PNGase) < truncated (endoS2) mAbs . aIM-MS was performed using the in-house built software ORIGAMI.^276^ Adapted with permission from ref (256). Copyright 2019 Royal Society of Chemistry.

One of the challenges in characterizing mAbs is that they inherently will be a mixture of slightly different proteins. If such analytes are examined in bulk, it may be difficult to differentiate the effects of a low abundance modification. The benefit of IM-MS assays is that they can examine mass isolated forms, but for mAbs and other large proteins, the mass differences between the proteoforms may hinder such experiments. An example of the structural insights provided by IM-MS experiments to determine proteoforms of mAbs and batch to batch effects was performed by Upton et al. in a study on three lots of an IgG1 mAb, herceptin, with different glycoform distributions,^256^ and at three different glycosylations: intact, partially, and fully deglycosylated.^343^ The rationale for examining the protein in these different stages of glycosylation was that the proteins become more homogeneous as the glycans are simplified or completely removed. To achieve this, the samples were treated with a mixture of the reagent IdeS, which cleaves just below the hinge region and EndoS2 (Figure 11a), which cleaves the bond between the two *N*-acetyl glucosamine residues of the *N*-glycans, resulting in a more homogeneous lowly glycosylated form. The second treatment involved a mixture of the reagent IdeS and PNGase (Figure 11b), which fully removed the *N*-glycan from the structure, resulting in a fully homogeneous deglycosylated mAb. CCS measurements from native IM-MS showed an increase in the conformational spread for each mAb, according to the extent of glycosylation, where intact CCS < deglycosylated CCS < endoS2 treated CCS. Similar experiments on the Fc-hinge fragment of the mAb, which possesses all of the glycan content with an overall lower molecular mass allowed a more detailed examination of the impact of glycosylation on the conformation of the CH2 and CH3 domains.^256^

The time required in aIM-MS analysis to produce robust and reproducible data that can be used to compare very closely related protein species provides an opportunity to characterize manufactured biotherapeutics, where glycosylation and different numbers and/or distribution of disulfide bonds can affect the overall structural stability of antibodies.^256,284^ Activated IM-MS experiments revealed lot-to-lot variations in herceptin even for the fully deglycosylated form.^256^ It was shown that the onset of restructuring required higher energy for the fully glycosylated forms. This suggests that conformational stability for mAbs is conferred by particular glycoforms. Native IM-MS was able to discern lot to lot variation; two out of the three lots had conformational profiles of high similarity but differed from the third, which had a wider conformational profile in all three levels of glycosylation (Figure 11d).^256^

#### 4.2.3 The Number of Disulfide Bonds and Their Bonding Patterns Affect the Structure of mAbs

**Figure 12:**
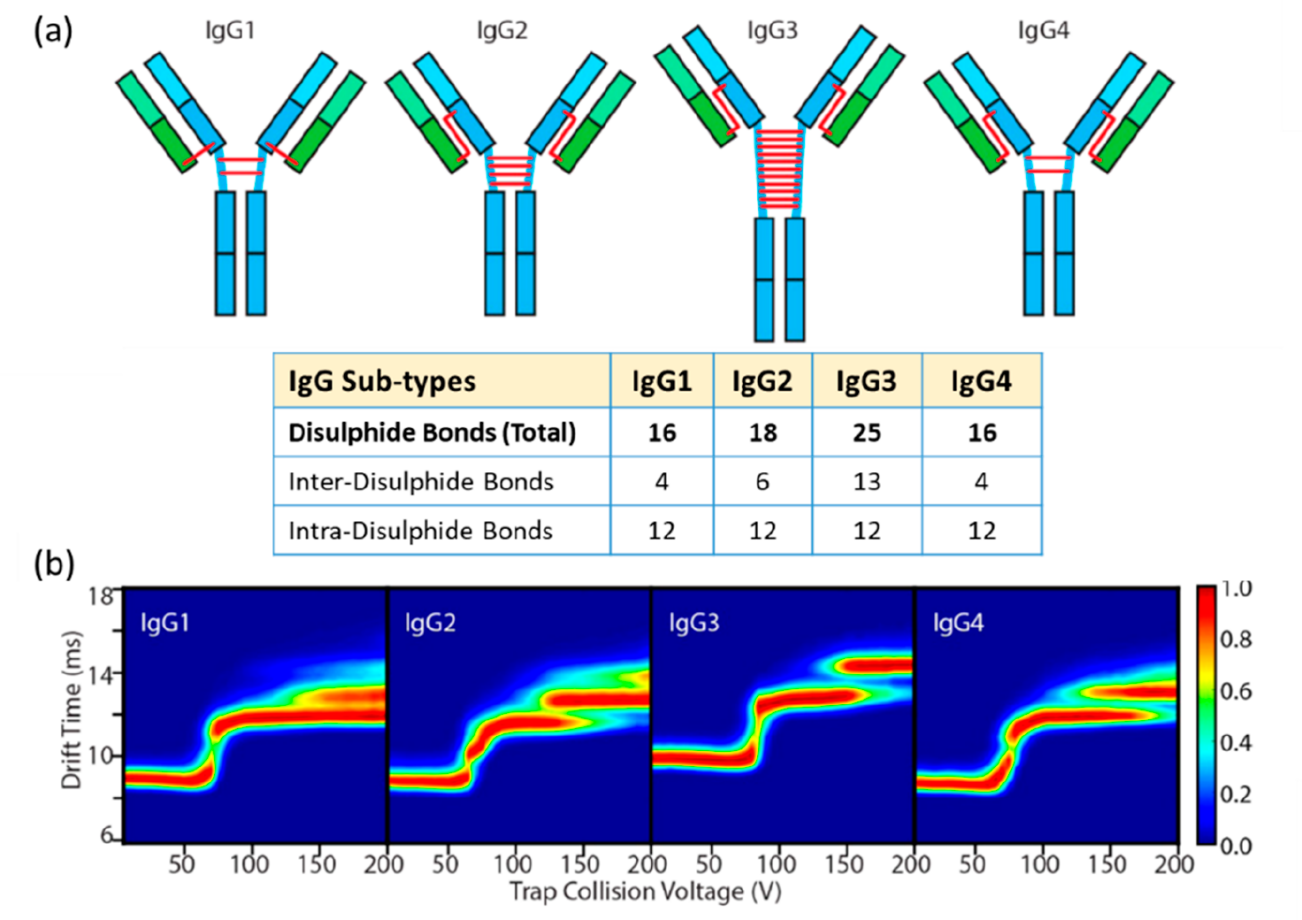
Collision-induced unfolding ion mobility mass spectrometry (CIU IM-MS) study on how the number and bonding arrangement of the interdisulfide bonds affect the conformational variation and stability of all four monoclonal antibody (mAb) classes. (a) mAb classes differ mainly on the number of disulfide bonds at the hinge region. IgG1 has a unique disulfide bond arrangement between the heavy (HC) and light (LC) chain of the Fab domain compared to IgG2,3 and 4. (b) Heat map comparisons show that IgG3 undergoes three structural transitions which are clearly separated. The initial folded state presents the longest drift time compared to the rest. Adapted with permission from ref (284). Copyright 2015 American Chemical Society.

Tian et al. used CIU to distinguish intact mAbs with different number and configuration of disulfide bonds (Figure 12).^284^ The four IgG subtypes (IgG1, IgG2, IgG3, and IgG4) are iso-cross-sectional species which differ by the number and/or patterns of their disulfide bridges. The first three isotypes, IgG1, -2, and -3 contain 4, 6, and 13 interdisulfide bonds, respectively. Despite these known chemical differences, native IM-MS of each results in highly similar CCS values (Figure 12a). CIU was deployed to study and distinguish between the four IgG subtypes based on their stability/unfolding profiles. IgG1, IgG3, and IgG4 each restructure via two dominant transitional states, whereas IgG2 presents four. For each IgG, these transitions occur at distinct activation energies. IgG3 contains 13 interdisulfide bonds and presented the longest initial ATD and possessed more abrupt transitions between each conformer, with little coexistence of states at any given energy. Each are found at later drift times than found for IgG1, -2, and -4. This observation is rationalized by considering that IgG3 has a more-constrained hinge region compared to the other IgGs.

This approach also can distinguish between IgG1 and IgG4, which have only marginally different disulfide bonding; each contains 4 intermolecular disulfide bonds with a slightly different binding configuration within the Fab domain (Figure 12a). Despite this, the DT centroids of the unactivated and activated conformers, the voltage required to induce the first transition, and the stability of the first transition state differs between the two (Figure 12b). These finding are similar to that previously reported by Pacholarz et al. using native IM-MS^211^ and variable temperature IM-MS,^344^ the latter of these being highly comparable to CIU experiments, another form of activated IM-MS.

The authors demonstrate how CIU is not just highly sensitive to the number of disulfide bonds but also to the bonding pattern within the mAb structure. This approach could be relatively easily applied for biosimilar or batch-to-batch comparisons in biotherapeutic development and production.

#### 4.2.4 Use of CIU/aIM-MS to Probe Ligand Binding

##### 4.2.4.1 cAMP-Dependent Protein Kinase (PKAc) Binding to Heat-Resistant Protein Inhibitor PKI

**Figure 13:**
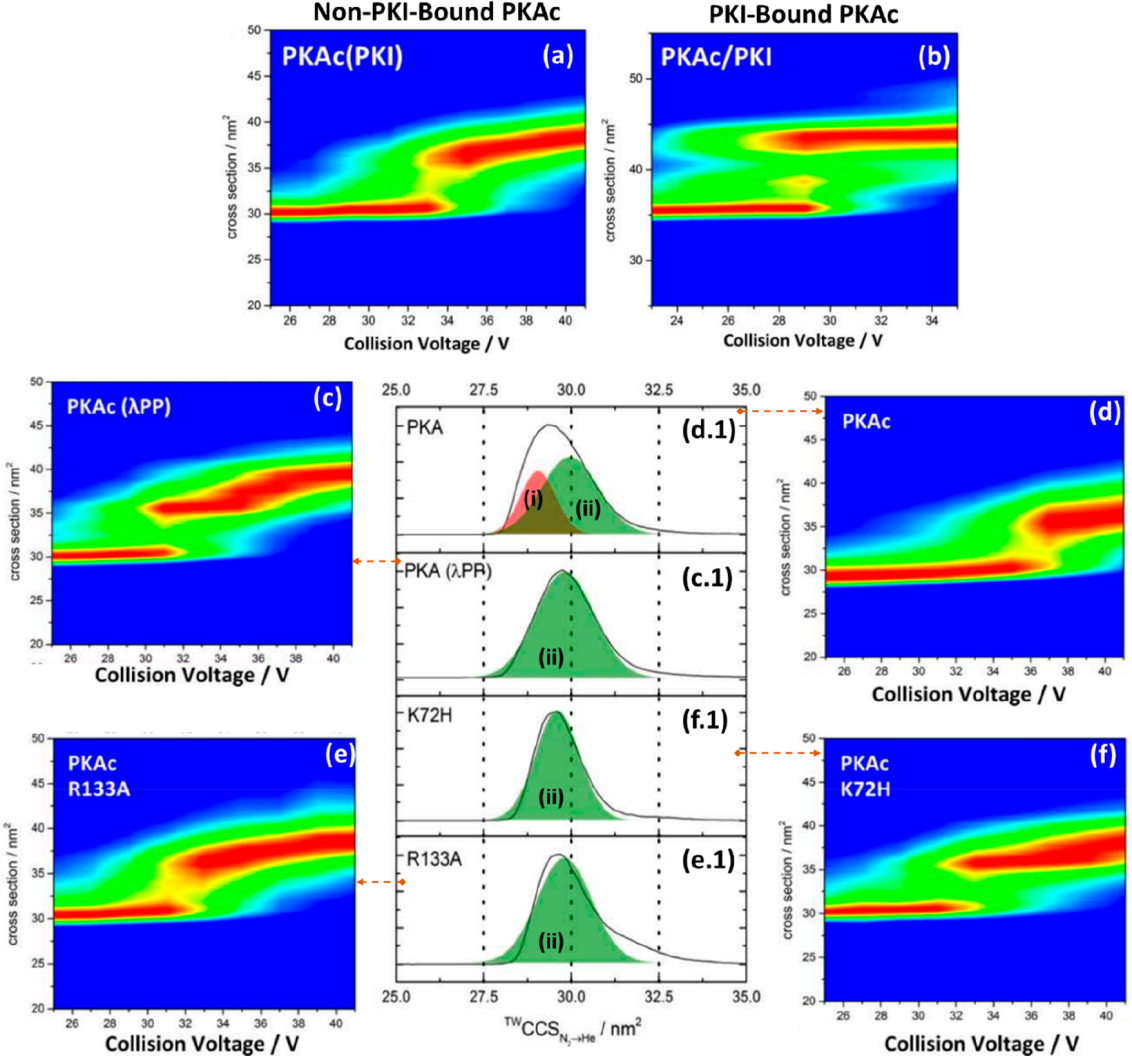
cAMP-dependent protein kinase (PKAc) undergoes two clean transition states when bound to the heat-resistant inhibitor (PKI) (b) and produces an elongated heat map and when unbound (a). The structural stability fluctuation of PKAc when treated with protein phosphatase, λPP (c), and two-point mutants, R113A (e) and K72H (f) was investigated. All three mutations destabilized PKAc as smaller collision voltages were used to induce unfolding (c,e,f) compare to the unmutated state (d). The ion mobility mass spectrometry (IM-MS) data (d.1) show the two main conformers of PKAc: (i) the conformer that can undergo binding with PKI (PKAc/PKI) and (ii) the elongated conformer that does not bind to PKI (PKac(PKI)). After the mutations, the IM-MS data (c.1, f.1, e.1) indicate the domination of the elongated conformer. Adapted with permission from ref (348). Copyright 2016 Portland Press under the CC BY 4.0 License, http://creativecommons.org/licenses/by/4.0/

CIU IM-MS has played a key role in examining the conformational stability induced by ligand and protein interactions, resulting in ligand–protein complexes.^266,280,282,345−347^ Byrne et al. used CIU IM-MS experiments as part of their method to examine how the structural and thermal stability of PKAc changes once it binds to the protein inhibitor PK (Figure 13a,b).^348^ They report how different variations of PKAc affect its ability to bind to the inhibitor by treating the wild-type (WT-PKAc) (Figure 13d) with two-point mutants, K72H (Figure 13f), R113A (Figure 13e), and with the protein phosphatase λPP (Figure 13c). It was apparent that the pure PKAc protein (Figure 13d) was the most stable and the three mutated monomers of PKAc (Figure 13e,f) undergo faster unfolding at lower collision voltages. They observed that the inhibitor (PKI) was not binding to the PKAc protein treated with the two-point mutants but would bind strongly to the WT. They conclude that the point mutants as well as the phosphatase protein treatments were destabilizing the protein monomer. With CIU experiments, they obtained two distinct intermediate states for the PKI-bound PKA complex, whereas the nonbound PKA complex presented an elongated drift profile (Figure 13a,b). The high activation voltage required for the dissociation of the PKA–PKI complex further supported ligand-dependent structural stability. Comparing the total conformer population of PKAc to the non-PKI-bound PKAc [PKAc(PKI)], it was possible to infer ligand-induced stability deterioration in the latter (Figure 13).

##### 4.2.4.2 Membrane Translocator Protein (TSPO) Bound to PI, PG Lipids, and Protoporphyrin IX (PPIX) Binders

**Figure 14:**
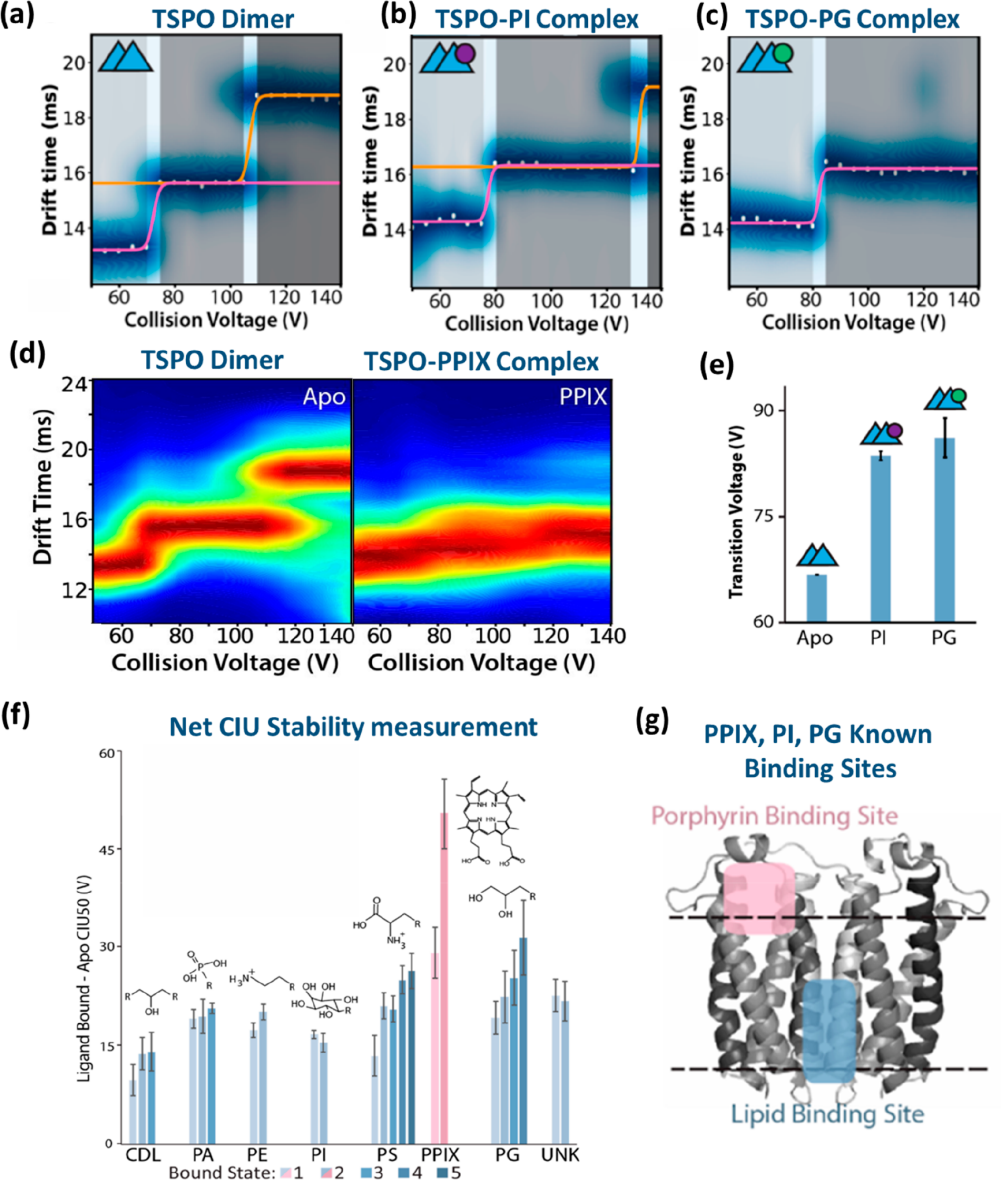
Stability studies using collision induced unfolding ion mobility mass spectrometry (CIU IM-MS) for (a) the pure TSPO dimer when bound (b) to PI lipids forming the TSPO–PI complex, (c) to PG forming the TSPO-PG complex, and (d) to propyrin (PPIX) forming the TSPO–PPIX complex. (e) The complex stability is quantified and error assessed across triplicates. It is evident that there is high stabilization in both lipid complexes compared to the pure complex (each triangle, TSPO monomer; green circle, PG lipid; purple circle, PI lipid). (f) Net CIU stability measurements for TSPO–ligand complexes (the difference between the inflection points for each sigmoid) indicate the increased stability of the TSPO–PPIX compared to the other lipid–TSPO complexes screened. An unidentified endogenous ligand (UNK) confers similar stability to the protein as other lipids examined. (g) Although the binding sites of the lipids and PPIX are well-known scant information, up until this report, was available regarding their stabilizing contribution to the TSPO structure. Adapted with permissions from ref (280). Copyrights 2019 American Chemical Society

Recently, Fantin et al. applied CIU IM-MS as part of their workflow to examine the stability of the membrane protein TSPO (36 kDa dimer) when complexed with phosphotinositol (PI) and phosphotidylglycerol (PG) lipids (in membrane) as well as protoporphyrin PPIX binders which bind to the extracellular loops (Figure 14).^280^ Limited information was available on the possible allosteric impact PPIX binders might impose on the membrane structure, although the binding sites of all three ligands examined were well-known (Figure 14g).

The CIU data showed that the pure TSPO dimer is less stable than either the TSPO–PI or TSPO–PG dimer complexes, for each unfolding occurs at higher collision voltages, with the TSPO–PG complex not proceeding to a more extended state unlike the pure and PI bound dimer (Figure 14a–c,e) highlighting the importance of specific lipids to stabilize the fold of this membrane protein. When the dimer TSPO is complexed with PPIX, it is further stabilized (even compared with the TSPO–lipid complexes) (Figure 14d). From net CIU stability measurements between the unbound (pure) TSPO dimer and a panel of TSPO interacting species, primarily lipids, they show that overall lipid interactions assist the stabilization of the protein albeit to different extents and that the extracellular drug stabilizes it the most (Figure 14f). They conclude that TSPO forms an even stronger complex with PPIX than with lipids and more generally that “TSPO–ligand complexes retain a strong memory of their native structure in the gas environment” again showing the capabilities of such measurements to monitor protein:small molecule interactions both in membrane and extracellular (Figure 14g).

#### 4.2.5 Use of CIU/aIM-MS for Disordered Assemblies and Amyloids

##### 4.2.5.1 UVR8 Photoreceptors

**Figure 15:**
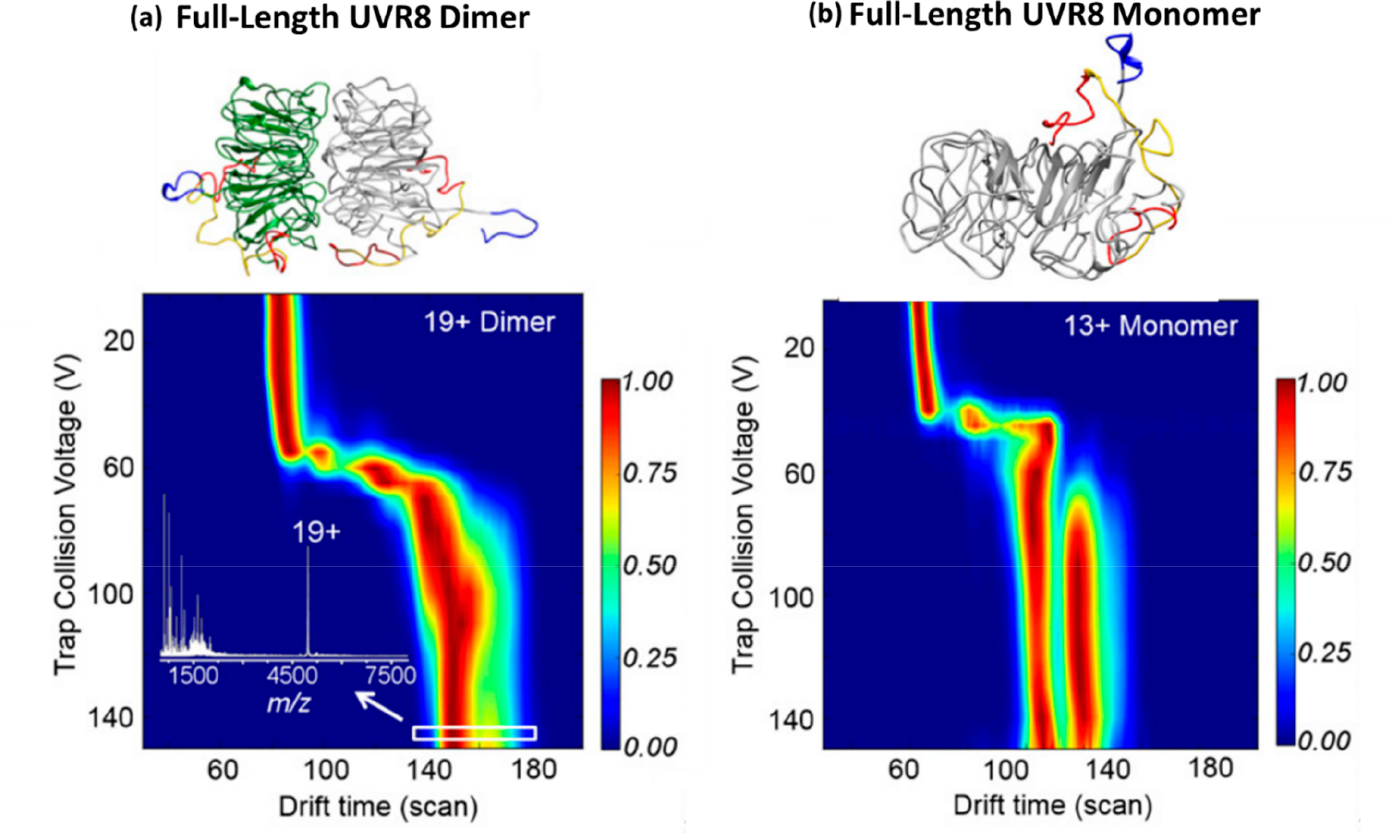
Activated ion mobility mass spectrometry (aIM-MS) heatmaps for the compact states of (a) the 19+ charge state full-length UVR8 dimer (dark) and (b) the 13+ charge state monomer (light), along with their respective structures obtained from trained gas phase molecular dynamic (MD) simulations. The core domain in the MD structures is in gray and green, the N-termini in dark blue, the C-termini in red, and the C27 region in yellow. (a) The dimer required ∼1000 eV to undergo a slow transition to a more extended yet still dimeric state, indicative of its high resilience also shown in collision induced dissociation (CID) experiments. The inset shows fragments primarily with sequence from the tails of the UVR8 along with the 19+ isolated dimer ion, but no intact monomer was observable. Adapted with permission from ref (270). Copyright 2019 PNAS under the CC BY 4.0 License, http://creativecommons.org/licenses/by/4.0/

For the UVR8 photoreceptor introduced in section 4.1.1, we have already described how CID was unable to dissociate the bioactive dimeric form.^270^ In an extension of that observation, activated IM-MS was used to investigate structural transitions that occur as the protein is activated up to and beyond the point where fragmentation of the N and C termini occurs. Initially, the dark state of the UVR8 dimer was collisionally activated and presented a compact low charge state which remains unaltered up to 1000 eV, before slowly transitioning into a more extended conformation (Figure 15a). The use of ORIGAMI acquisition and processing software permitted tracking of the conformations of the protein and its fragments during activation.^270,276^ Similar experiments were used to examine conformational transitions in the light activated monomer (Figure 15b). From comparing CCS values from IM-MS measurements to those obtained from directed MD simulations, which were trained by the experimental data, it was demonstrated that primarily imparted by the disordered termini, the UVR8 photoreceptor exists in numerous conformational families as both a dimer and a monomer. The conformational spread found in the native IM-MS data was also achievable using aIM-MS from compact conformers of the dimer, showing how dynamic this functional protein is, adding to the structural work previously performed which had considered the core domain as a sufficient proxy for the native form.^270,273^

### 4.3 Surface-Induced Dissociation (SID)

Surface induced dissociation (SID) is performed by accelerating a given ion toward a surface which is usually coated with a polymeric material to dissipate energy.^349,350^ A more detailed description of the SID process is beyond the scope of this review, and we direct the reader to recent publications.^252,264,351−354^ Briefly, the impact with the surface causes a rapid increase of the ions’ internal energy compared to the larger number of low-energy collisions in CID. By tuning the impact energy and even the surface coating, it is possible to achieve efficient and selective fragmentation for a range of analytes. Wysocki and co-workers have incorporated SID with IM-MS for a variety of studies including confirmation of the topology of subunits in designed protein complexes, distinguishing isobaric ions by producing highly different SID fragmentation profiles, studying the dissociation of noncovalent complexes, and predicting protein complex structures.^253,264,268,351,355−357^ Due to the speed of energy transfer in these single higher-energy collision, biomolecules are less prone to undergo restructuring. As a result, SID promotes the generation of compact subunits with low charge and symmetric charge partitioning that are more reflective of the native topology than for classical low energy CID.^354,357^ Below, we highlight some examples where SID IM-MS has been used to study simple biomolecules, homomeric complexes, and heteromeric complexes.

#### 4.3.1 Simple Approaches

##### 4.3.1.1 DesArg1 and DesArg9 Bradykinin Peptides

**Figure 16:**
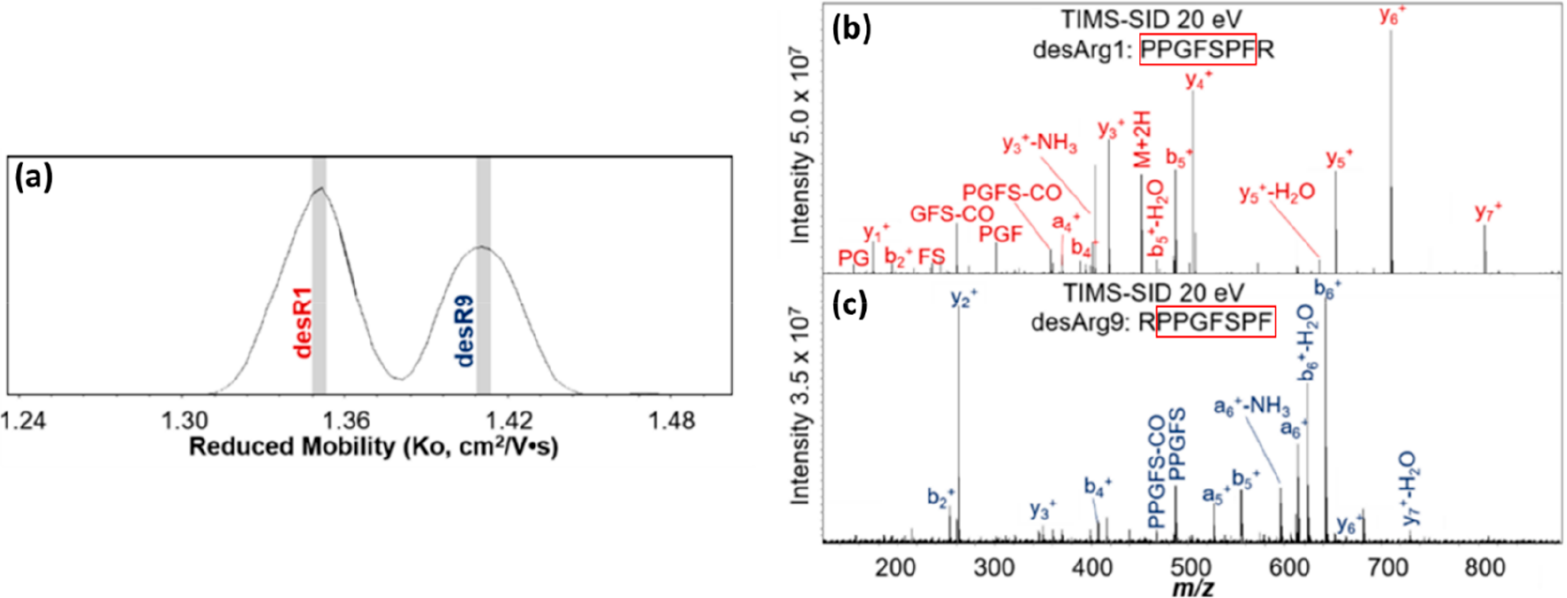
Fragmentation patterns produced using the surface induced dissociation trapped ion mobility spectrometry (SID-TIMS) for a 1:1 desArg1:desArg9 bradykinin peptides mixture. (a) Poor separation of the two isobaric peptides, desArg9 and desArg1. (b) Fragmentation pattern of desArg1 and (c) fragmentation pattern of desArg9 using SID-TIMS. Full identification of the two isobaric ions was achieved after two highly unique fragmentation patterns were generated. Adapted with permission from ref (253). Copyright 2021 American Chemical Society.

Starting from a simple, effective example, Panczyk et al. coupled SID with TIMS to investigate the fragmentation patterns obtained by desArg1 and desArg9 bradykinin peptides.^253^ DesArg1 (lacking the N-terminal) and desArg9 (lacking the C-terminal) are isobaric, making their separation with conventional MS approaches difficult (Figure 16a). With SID, each peptide produced substantially different SID fragmentation spectra allowing their identification, which was later confirmed with TIMS (Figure 16b,c).^253^ This work demonstrated the feasibility of coupling SID with TIMS ion mobility and as with other forms of IM, the opportunity for its use for more challenging structural biology problems.^357^

#### 4.3.2 SID Working in Concert with Protein Design

##### 4.3.2.1 Streptavidin, Tryptophan Synthase (TS) Assembly Pathways

**Figure 17:**
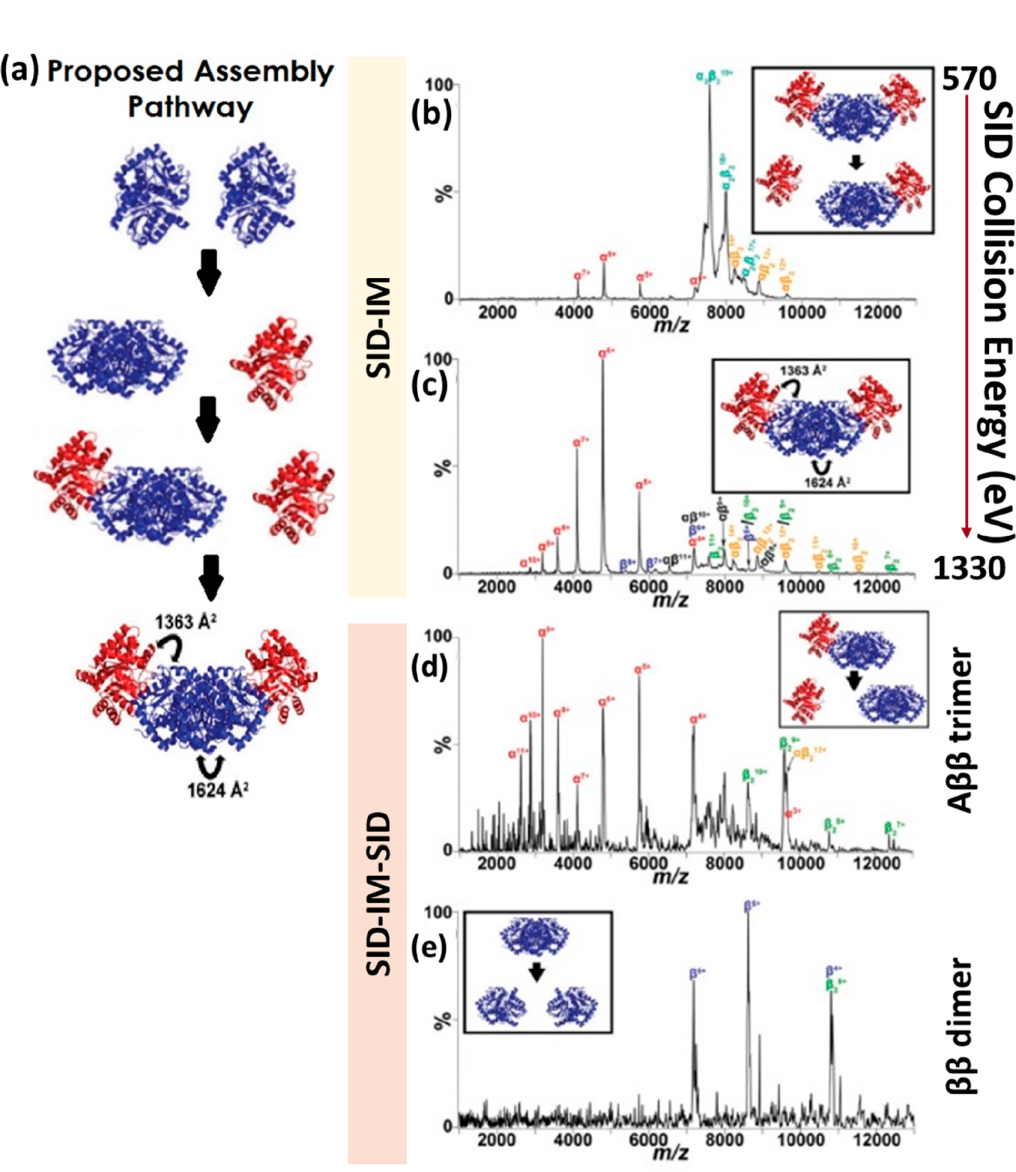
Predicted assembly pathway derived from two different arrangements of surface induced dissociation ion mobility experiments (SID-IM and SID-IM-SID). (a) The β monomers (blue) interact to form the ββ dimer, after which the two alpha monomers (red) bind to each of the β interfaces to form the tetrameric complex. The a/β and ββ interfacial surface areas were calculated to be 1363 Å^2^and 1624 Å^2^, respectively. SID-IM was used to dissociate the 19+ tetramer and study the dissociation pathway using both (b) low (570 eV) and (c) intermediate (1330 eV) SID collision energies. The dominant dissociation pathways are shown in the insets. Lower energy (570 eV) SID-IM dissociate the smaller interfacial surface area, whereas intermediate (1330 eV) generated ββ dimers. SID-IM-SID was then used to further dissociate (d) the αββ trimer (yellow) and (e) ββ dimer (green) produced from the SID-IM using higher collision energies (2280 eV) in the second SID device. The color-coding of the subunits resulting from the SID fragmentation are β monomer (blue), a monomer (red), ββ dimer (green), aβ dimer (black), aβ_2_ trimer (yellow), and a_2_β_2_ tetramer (turquoise). Adapted with permission from ref (355). Copyright 2015 The Royal Society of Chemistry.

In foundational study, Quintyn et al. used SID-IM-SID to study the assembly pathways of streptavidin, a homotetramer composed of dimers, and tryptophan synthase, which is found with a linear αββα structural arrangement.^355^ They constructed a novel set up with two SID devices, one on each side of the IM cell of a Waters Synapt G2-S mass spectrometer. In initial experiments they were able to generate subcomplexes with the first SID device, separated them in the IM cell to gain structural information post dissociation and then further dissociated them in the second SID device into smaller subunits. In this way, they were able to dissect the quaternary structure of these homo and hetero complexes and then validate a hypothesis that, at low energies, dissociation in complexes proceeds between subunits with the smallest interface area and that higher energies are required to achieve dissociation between subunits with larger interfaces (Figure 17).

For both the homo and hetero complexes, this work showed that the energy required for dissociation increases with the area of the interface taken from crystallographic evidence. The interfacial surface area was theoretically enumerated using the software PISA^358^ and for streptavidin was 1363Å^2^ for the α/β interface and 1624Å^2^ for the β/β interface (Figure 17a). From the SID-IMS, they observed initial dissociation of the α/β interface, at low SID collision energies (570 ev), producing an alpha monomer and the remaining αββ trimer (Figure 17b). This dissociation site preference validates the interface/collision energy hypothesis from the calculated interfacial areas; the αβ interface is smaller than that of ββ. By increasing the SID energy to an intermediate level (1330 eV), they observed ββ dimers implying that the two subunits are connected in the quaternary structure (Figure 17c). No αα dimer was observed and in combination with the αββ trimer, they concluded that the ββ dimer is surrounded by the α subunits. To gain further information on the quaternary structure the αββ trimer and ββ dimer were further dissociated in the second SID device postseparation in the IM cell (SID-IM-SID) (Figure 17d,e). From the dissociation of the dimer, two β monomers were acquired as expected (Figure 17e).

The assembly pathway of the TS heterotetramer was thus proposed to be completed in three steps: (1) the ββ interface is the first to form, (2) an α monomer interacts with one of the ββ dimers to form the αββ trimer, and (3) another α monomer interacts with the available β side of the trimer to complete the αββα tetrameric structure (Figure 17a). This work highlights how SID-IM-SID can be used to predict the assembly and topology of complex homo- and heteromeric complexes that could adopt multiple structural arrangements.^355^ It also suggests that it is possible to preserve native protein interfaces in vacuo and that SID IM-MS experiments can be used to ensure that they have been with interactive control of source and or solution conditions. A similar approach was adopted by Black et al. using UVPD coupled to IM-MS (see below).^257^

##### 4.3.2.2 Using SID IM-MS to Confirm Predicted Topologies of Designed Dodecameric Protein Assemblies

**Figure 18:**
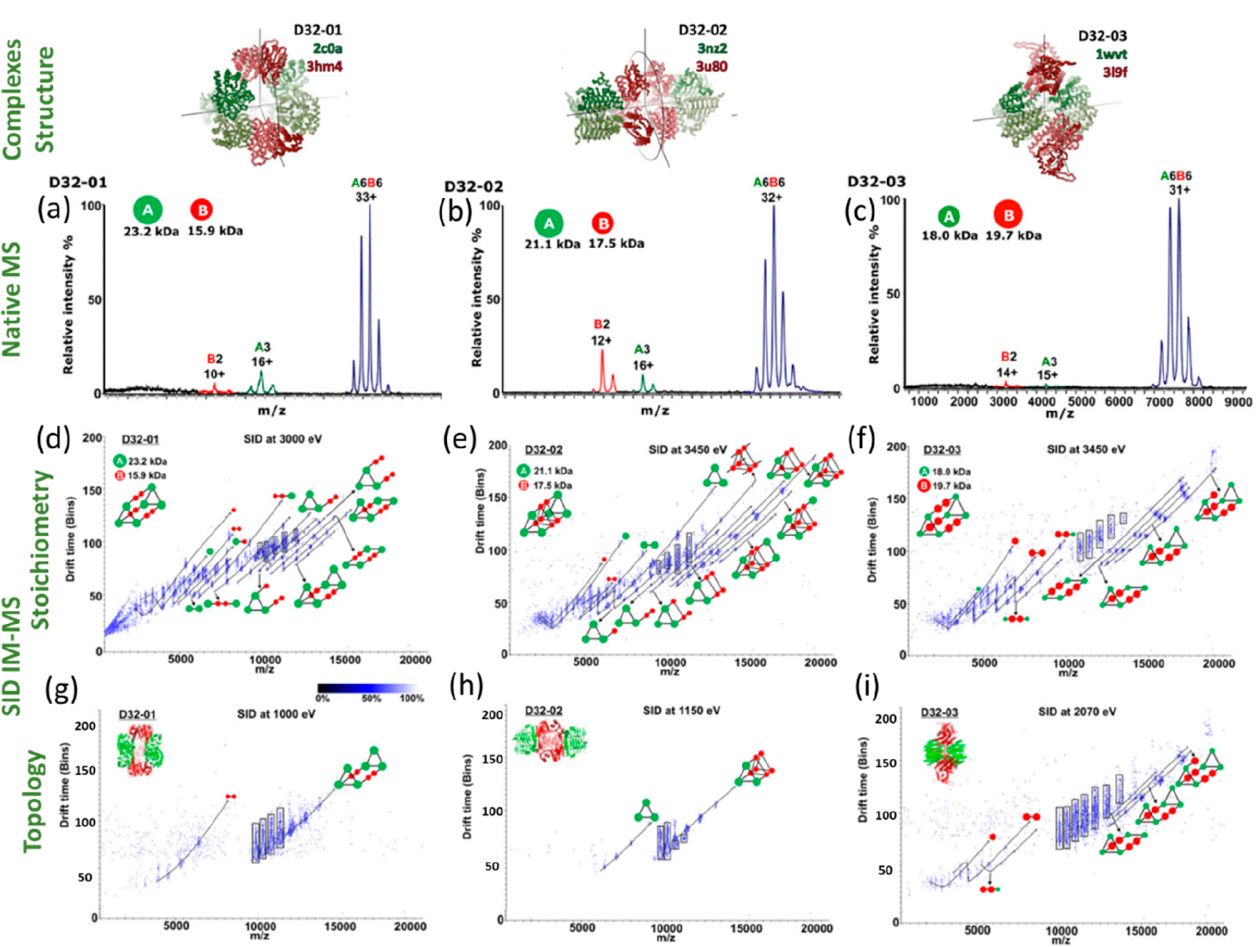
Confirmation of the intersubunit connectivity and topology of a designed heterododecamer protein complex sample using SID IM-MS and native MS. Native mass spectra of (a) D32-01 complex, (b) D32-02 complex, and (c) D32-03 complex samples. “A” is a trimer, “B” is a dimer, and A6B6 denotes the heterododecamer complexes. The complexes’ structure and wild-type scaffold PDB ID is shown on top of each spectrum. Surface induced dissociation ion mobility mass spectrometry (SID IM-MS) of mild- SID energy (d–f) and low- SID energy (g–i). SID IM-MS data are shown for the selected ions (d) 9527 *m*/*z*, 25+, D32-01 at SID of 3000 eV, and (g) 1000 eV for (e) 10482 *m*/*z*, 23+, D32-02 at SID of 3450 eV, and (h) 1150 eV and for (f) 10482 *m*/*z*, 23+ D32-03 at SID of 3450 eV and (i) 2070 eV. Charge state series (black continuous lines) from the SID products were used to calculate the subunits’ mass and thus identity. Charge-stripping ions are highlighted in the black rectangles. Only the subunits identified with great confidence are shown. Each A6B6 complex is thus composed of three dimers (A6, two red connected dots) and two trimers (B6, green triangles). Adapted with permission from ref (356). Copyright 2018 U.S. Academy of Sciences.

In recent work, Sahasrabuddhe et al. outline more advanced applications of SID in combination with IM-MS.^356^ One of the main motives behind the choice of SID over any other activation technique is that it is possible to tune the activation conditions such that noncovalent dissociations are favored, enabling the release of native-like subunits (Figure 18). Here the authors considered three heteromeric dodecamer protein complexes consisting of three dimers and two trimers which had been designed in silico to possess very different topologies. The main difference between these complexes was the arrangement of different subunits around the helical axis. This resulted in different interfaces and resultant protein–protein interactions (PPI) between the dimers and trimers along with slight mass fluctuations.

SID IM-MS was used to determine the stoichiometry and organization of the different subunits of the designed complexes and to compare experimental findings with those predicted by computational design.^356^ As a first step, using IM-MS, they evaluated the CCS values of each complex (D32-01, D32-02, and D32-03) and compared them with computational CCS derived from the models. The CCS deviations were 7%, 0.2%, and 11%, respectively, with the largest one associated with some collapse in the gas phase. For the topology and PPI studies of the different subunits, SID was incorporated into the IM-MS experiments in which the different complexes were exposed to different acceleration voltages. They found that D32-01 complex ejects a single dimer at low acceleration voltages and infer that the interaction between the dimeric and trimeric subunits is weak (Figure 18g). Similar behavior was detected for the complex D32-02, which ejected a trimer (Figure 18h). The dissociation behavior of both complexes was in good agreement with the computational predictions made on the strength of the protein–protein interfaces.

Interestingly, the last complex, D32-03 was experimentally found to be more stable, which did not agree with the prediction (Figure 18i). Very high acceleration voltages (∼2070 eV) were required to induce any dissociation, and even then, only dimer subunits were detected (Figure 18i). The authors suggest that the large energy required to trigger dissociation caused structural rearrangement in the gas phase preventing further dissociation. The authors conclude that low-energy SID activation can cause the dissociation of weak PPIs, with small interfaces, whereas higher-energy SID activation favors the dissociation of the larger interfaces or even dissociate dimers into their monomer counterparts (Figure 18d–f). SID IM-MS experiments helped confirm that the complexes designed indeed consisted of the dimeric and trimeric subunits and each monomer of the trimeric units binding to the dimer in the center.

### 4.4 Ultraviolet Photodissociation (UVPD)

UVPD can be operated in a similar fashion to SID such that energy is transferred onto the analyte rapidly (nanomicro seconds), whereas in ETD/ECD and CID, the same amount of energy will be deposited over a longer time period (micro- to milliseconds).^258,359^ The time taken to activate and break an interaction for SID has been predicted to be between 10^–10^ and 10^–14^ s, and for UVPD performed with a wavelength of ∼193 nm the time scale for bond activation is between 10^–15^ and 10^–16^ s.^262^ In UVPD in vacuo, high-energy photons can be focused along or across the analyte beam in quadrupole ToF instruments or into the ion cloud in ion traps.^257,359−364^ Adsorption of a single UV photon of λ < 215 nm is sufficient to cause electronic excitations in the protein backbone and thus dissociation as certain bonds are efficient chromophores at these deep UV wavelengths. The common use of specific wavelengths for UVPD allows this method to be more targeted to certain bonds than other activation methods.

UVPD has had considerable application in structural biology and has also been applied in combination with IM-MS. Adaptation of UVPD on a mass spectrometer requires some instrumentational modifications/considerations. In a series of papers, Bellina and Theisen et al. described the coupling of a UV laser with a Waters G2S Synapt.^254,365,366^ For this approach to be successful, the laser and ion beam needed to overlap in the transfer ion guide. This was achieved by modifying the IM-MS instrument so that the laser is directed through a window in the ion source through the instrument to a beam dump created by locating a 45° angled mirror in the pusher plate of the ToF. In this configuration it is possible to perform photodissociation in different regions of the mass spectrometer, in the source, the trap, or the transfer ion guides that flank the IM cell.^255,365^ In any given experiment it proved beneficial to trap the ions to maximize photointeraction and to control this using WRENS, Waters Research Enabled Software, which allows additional controls of the Synapt hardware.

#### 4.4.1 UVPD to Achieve Complete Coverage of Intact Proteins up to 29 kDa

##### 4.4.1.1 Pin1 (Q13526) Protein, Ubiquitin, Myoglobin, and Anhydrase

**Figure 19:**
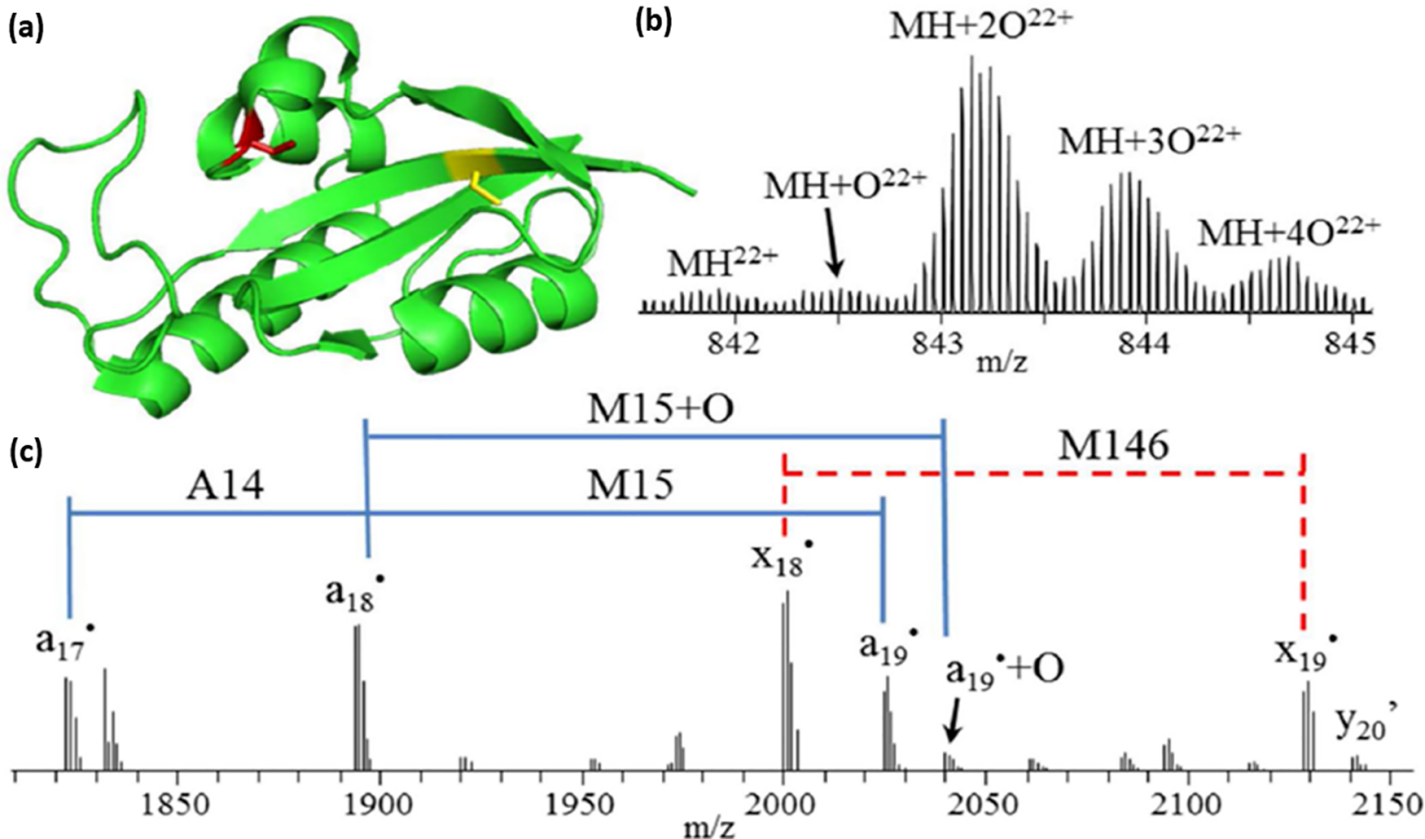
(a) The crystal structure of the Pin1 protein on which the red strand is the Cys113 oxidized residue and the yellow strand is the Cys57 nonoxidized residue. (b) The intact mass spectrum of the protein is presented, clearly showing the doubly and triply oxidated species which are the most abundant peaks. (c) From the UVPD mass spectrum, the alpha fragmentation ions with the Arg14Ala (A14) and oxidized Met15 mutations were identified. Adapted with permission from ref (360). Copyright 2013 American Chemical Society.

Without using IM, in foundational studies Shaw et al. report almost complete sequence coverage of numerous intact proteins; ubiquitin (8.5 kDa), myoglobin (17 kDa), and carbonic anhydrase (29 kDa), using a single 5 ns pulse of a 193 nm UVPD laser beam.^360^ For myoglobin, UVPD provided better sequence coverage (93%) than other fragmentation techniques. For the other proteins, UVPD gave 100% coverage for ubiquitin and 87% for carbonic anhydrase. In addition, UVPD allowed the localization of mutations at individual residues in the protein Pin1 that has been associated with the progression of Alzheimer’s and cancer (Figure 19a).^360^ Sequence coverage of 96% allowed the authors to identify deoxidation of Cys113 as the major product and confirm the lack of oxidation of Cys57, which agreed with crystallographic evidence. Further work mined the fragments and permitted the identification of a single residue mutation, Arg14Ala, and partial oxidation of Met15 (Figure 19c).^360^ Although direct relations between their finding and Alzheimer’s disease cannot be drawn, it is evident that top-down mass spectrometry, which allows precise selection of the precursor ion, coupled with UVPD can be used to study proteoforms implicated in such diseases even at a single residue level.

#### 4.4.2 UVPD Coupled with IM-MS to for Conformation Dependent Top-Down Analysis

In a recent study, Black et al. used multiplexed fragmentation strategies (UVPD, CID) and multivariant analysis (MVA) to characterize native protein structures.^257^ The study focused on multiple model proteins of increasing structural complexity, namely, cytochrome c, ubiquitin, and the multimeric proteins conconavalin a and human hemoglobin. A 213 nm laser beam was introduced via a CaF_2_ source block window to their in-house modified IM-MS instrument and was aligned with the ions’ pathway.^365^ The mass selected ions were accumulated (2 s) in the trap region, and once they reached intensities of ∼2e^3^, they were photoactivated with the laser beam. The photoactivated products were then separated in the IM cell and optionally activated in the transfer region post the IM cell by increasing the CE voltage. The multimeric proteins were not trapped before photoactivation, this had little effect on the ATD obtained for ubiquitin, which indicates that the protein conformations are not altered by the trapping under these conditions.

**Figure 20:**
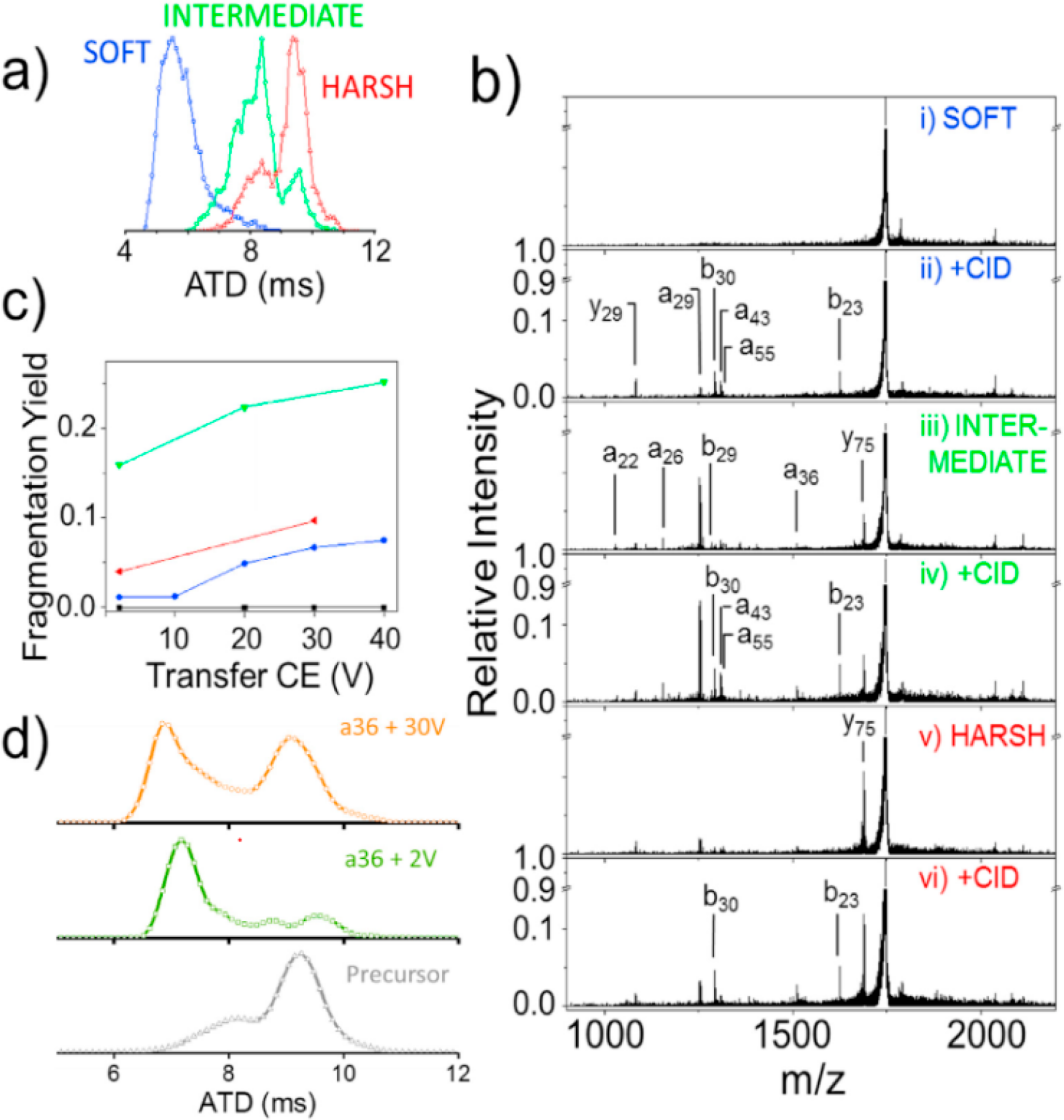
Ultraviolet photodissociation ion mobility collision-induced dissociation (UVPD-IM-CID) experiments performed *z* = +7 cytochrome c ion. (a) Arrival time distributions (ATD) of the +7 ion at three source conditions: soft (cone 10 V), intermediate (cone 85 V), and harsh (cone 120 V). Soft ionization conditions lead to a single compact conformer, which becomes more extended as the conditions become harsher (intermediate and harsh). (b) UVPD spectrum with soft source conditions without CID (i), with additional transfer collision energy (CE) of 30V (ii), UVPD spectrum with intermediate (85 V) source conditions and without CID (iii), with additional transfer cell collision energy (CE) of 40 V (iv), UVPD spectrum with harsh (120 V) source conditions without CID (v), with additional transfer cell collision energy (CE) of 30 V (vi). (c) Fragment yield of the α-type fragments as a function of the transfer collision energy applied for the control, only CID applied (black), soft (blue), harsh (red), and intermediate (green) source conditions. (d) Arrival time distribution (ATD) of fragment a36 produced from harsh source conditions and further exposed to transfer collision energies (CE) of 2 V (green) and 30 V (orange). The precursor with no transfer activation is shown in gray. Reproduced with permissions from ref (257). Copyright 2019 Elsevier BV.

The approach followed by Black et al. emphasizes the fragmentation pattern differences between CID and UVPD techniques that highly depend on the conformation of the precursor ions. They observed that collision activation of photoactivated products in the transfer regions, lead to detection of noncovalently linked fragments in both extended and compact conformers. With manipulation of the source cone voltage (Figure 20a), they induced multiple analyte conformers. A range of in source activation conditions were examined by setting the cone voltage to 10 V (soft), 30 V (intermediate), and 85 V (harsh) conditions (Figure 20a). Hence, with the incorporation of both CID and UVPD, they were able to (Figure 20b) map fragments to the IM data and facilitate a conformer-based univariate data analysis approach, which provided ab initio models to predict stability of the proteins.^257^

### 4.5 Electron Transfer Dissociation or Electron Capture Dissociation (ETD or ECD*)*

ETD/ECD are related techniques that fragment single bonds by electron mediated reactions and can be applied in a way that preserves PTM as well as noncovalent interactions. In both methods, bond cleavage follows after a protonated analyte captures an electron from the electron carrier.^367−371^ The main difference between the two is the source of electrons. In ECD,^369^ electrons are conventionally sourced from a heated filament in vacuo, whereas in ETD,^370^ precursor cation fragments after reacting with a preionized anion, such as fluoranthene, commonly applied in source.^372^ ECD was discovered serendipitously by Mclafferty and co-workers in an attempt to perform UVPD within an FT-ICR cell, their laser was striking metallic surfaces and causing the production of electrons.^369^ The precursor cation interacts with the electron carrier, which results in reactions by the electron within the peptide chain and the formation of unstable radical cations. When applied to proteins, the resulting products of ETD/ECD are reduction of the precursor and *z* and *c* fragment ions following backbone cleavage along the N–Ca bond. The limitation of these methods are that ETD/ECD is most effective for precursor cation of high charge states, which limits its deployment to native protein complexes that tend to be produced with relatively low charge state distributions. Until recently ECD was only possible on FT-ICR instruments, which also limited its uptake, but now an efficient ECD cell can be retrofitted to both QToF and orbitraps.^373−380^ Much of the usage of these electron mediated methods has been for peptidecentric studies to provide complementary fragment information to that achieved by CID, however, in native mass spectrometry approaches, they have had more selective application for highly detailed structural investigations for both proteins^119,370,372,381−387^ and other biopolymers.^388−390^ As with UVPD, ECD and ETD provide an opportunity to examine which parts of a given protein sequence are released from the main fold following activation or natively within dynamic complexes, such studies are often well supported with IM-MS measurements, and here we highlight some pertinent examples.

#### 4.5.1 Apolipoprotein E(apoE) Tetramer

**Figure 21:**
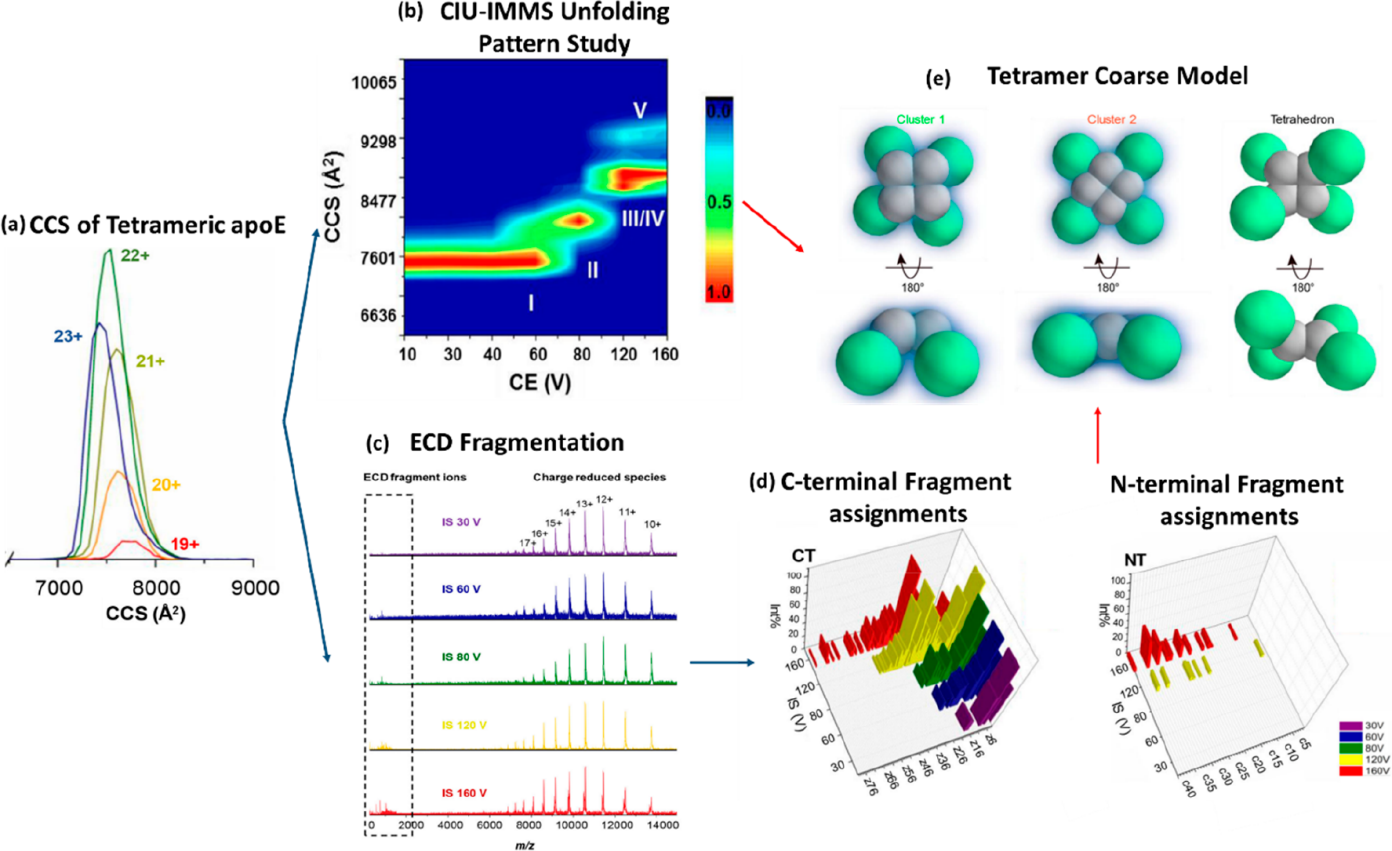
Summary of the workflow followed to improve and develop the low-resolution ion mobility mass spectrometry (IM-MS) data and coarse model for the tetrameric apoE. (a) CCS distributions of the tetrameric apoE species were obtained to identify the conformational spread, (b) the tetrameric protein was exposed to both CIU experiments to examine its unfolding patterns, and (c) ETD/ECD was applied to examine which of the two terminals undergoes unfolding first. For the ETD/ECD fragmentation, the molecule was activated in the same fashion as the CIU experiments by increasing the skimmer voltage (d). The N-terminal (NT) and C-terminal (CT) fragments produced by the ECD experiment were then assigned and (e) all considered the coarse model was improved. Adapted with permission from ref (384). Copyrights 2019 American Chemical Society.

Following IM-MS analysis, Wang et al., used ECD experiments to test a hypothesis regarding the unfolding of the tetramer of the apolipoprotein E protein apoE.^384^ The width of apoE CCS distributions indicated that the tetrameric species presents a single conformer, which is slightly broader than for the monomeric species and narrower than proteins of a similar mass (Figure 21a).^384^ They combined CIU and ECD top-down experiments to start to unfold the protein and sequence flexible regions as well as map exposed surfaces of the protein.^384^ The dominant fragments from the activated tetramer were mapped to the C terminus, which supported a *C*4 symmetry estimate of the structure of the tetramer (Figure 21c,d).^384^ Experimental findings were then used to validate and improve a low-resolution coarse-grained model of protein unfolding^384^ Not only does this work highlight the combination of ECD coupled with IM-MS, it also shows how IM-MS techniques can be integrated with coarse-grained models to provide candidate geometries for dynamic protein complexes.

#### 4.5.2 Lymphotactin Protein, Ltn

**Figure 22:**
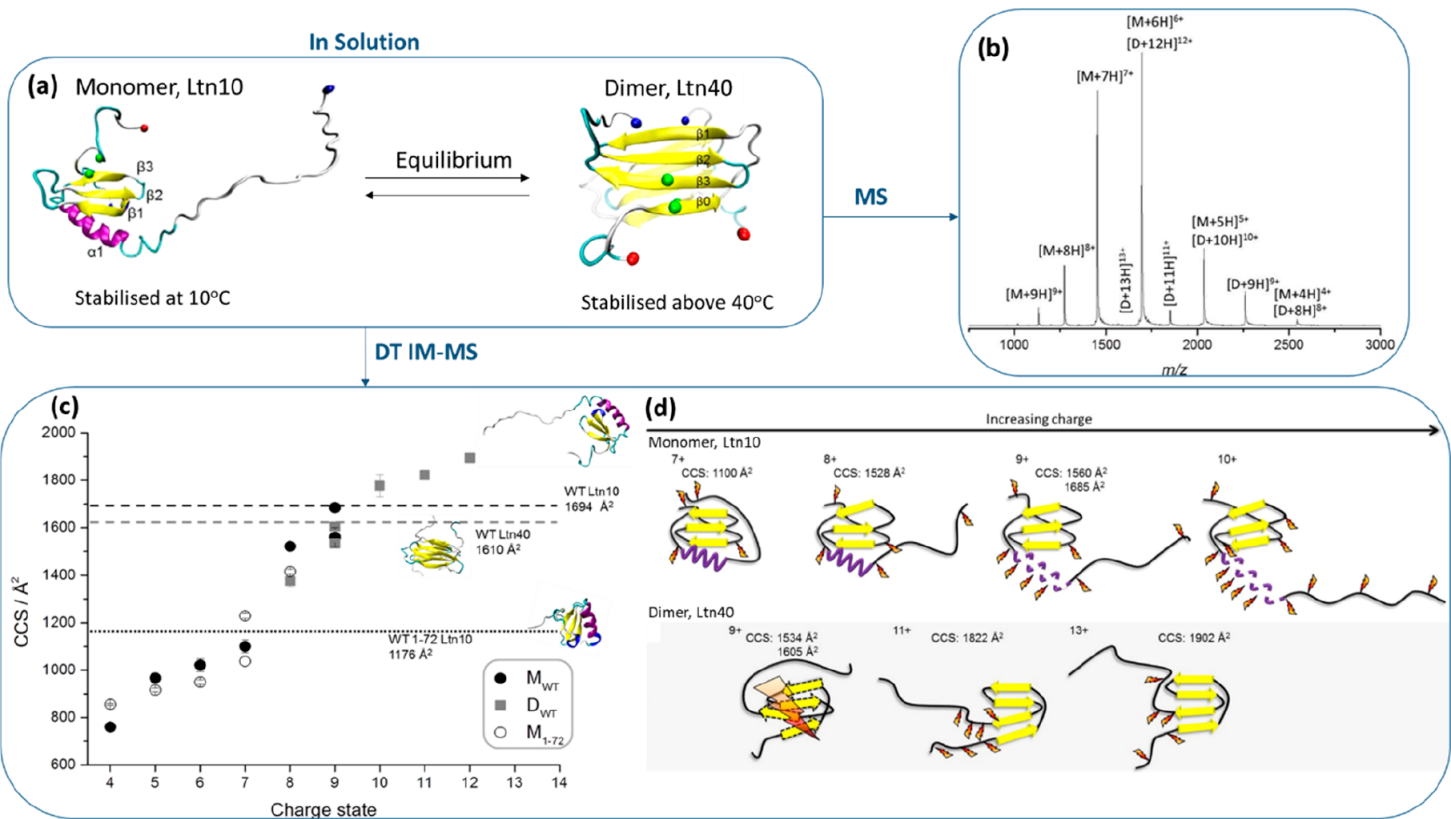
(a) Metamorphic protein, lymphotactin (Ltn) is found in equilibrium between its monomeric and dimeric states in solution. The N-terminus is highlighted in red spheres, the C-terminus is highighted in blue spheres, the location of the disulfide bonds is highlighted in green spheres, core β-sheets are highlighted in yellow, and α-helixes are shown in pink and purple. (b) By using native-MS, the sample heterogeneity was established. (c) From the drift time ion mobility mass spectrometry (DT IM-MS) data, a comparison between the experimental collision cross section (CCS) values of the monomer (solid circles), dimer (solid squares), and truncated Ltn1–72 (empty circles) were compared to the theoretical CCS values. Dashed lines are the theoretical CCS from the PDB files 1J90 and 2JP1. The dotted line is the theoretical CCS of the truncated structure from the PDF file 1J90 considering only residues 1–72 (d). The orange lightning symbols denotes the areas where significant ETD/ECD fragmentation was observed, dashed lines represent regions with not significant secondary structure. Destabilization of the C-terminal resulted in unravelling of the α-helix and consequently its fragmentation. ETD/ECD fragmentation for the compact structures was successful only on the terminal sides, where secondary structures and salt bridges were not present. Indeed, the α-helixes and β-sheets and regions with salt bridges are highly stable. Adapted with permission from ref (244). Copyright 2014 American Chemical Society.

Lymphotactin (Ltn) is a protein that is usually found in equilibrium between its monomeric (Ltn10) and dimeric (Ltn40) states in solution (Figure 22a). Results from top down ECD on monomeric, dimeric, as well as a truncated form (excluding the disordered tail, Ltn1–72) of lymphotactin were integrated with data from DT IM-MS to examine the conformational dynamics of this mesomorphic protein (Figure 22c)^251^ following the approach taken by Bruker and co-workers^391^ with helical proteins, where they asserted that lower fragmentation occurs from regions rich in secondary structure.^244^ For Ltn, Harvey et al. combined ECD and IM data also to evaluate how protein’s secondary structure is retained in the gas phase, and in particular if β-sheets are retained.^244^

DT IM-MS experiments revealed flexibility in the monomer (Ltn10), whereas the dimer (Ltn40) was more compact and structurally more homogeneous. By performing ECD on charge state selected forms of Ltn in an FT-ICR MS, fragment maps were used to infer the stability of the secondary structural regions. Lower fragment yield from lower charge states, which also had lower CCS, was attributed to the disordered tails of Ltn being wrapped around the folded core (Figure 22d) and a large number of noncovalent interactions preventing appreciable fragment loss. In higher charge states, with higher CCS values, it was assumed that the tail was now no longer closely associated with the β-sheet core Still higher charge states indicated further conformational disruption, where it was hypothesized that the α-helix started to unravel. These assertions from experiment were supported by calculations from NMR structures^244^ and the increased yield of fragments from the α-helical regions for higher charge states. It was found for LtN that the β-sheet core remained intact even in the higher charge states and was deemed the most stable, followed by the α-helix regions and the N- and C-termini.^244^ They also observed that salt bridges enhance structural stability in the gas phase, as little to no fragmentation was achieved at regions where they were present. A favorable fragmentation site was near residue 52, which in the NMR structures corresponded to a region with minimal secondary structure nor salt bridges.

#### 4.5.3 Use of Electron Transfer with No Dissociation (ETnoD) IM-MS to Assess the Impact of Charge Reduction on the Protein Conformation

**Figure 23:**
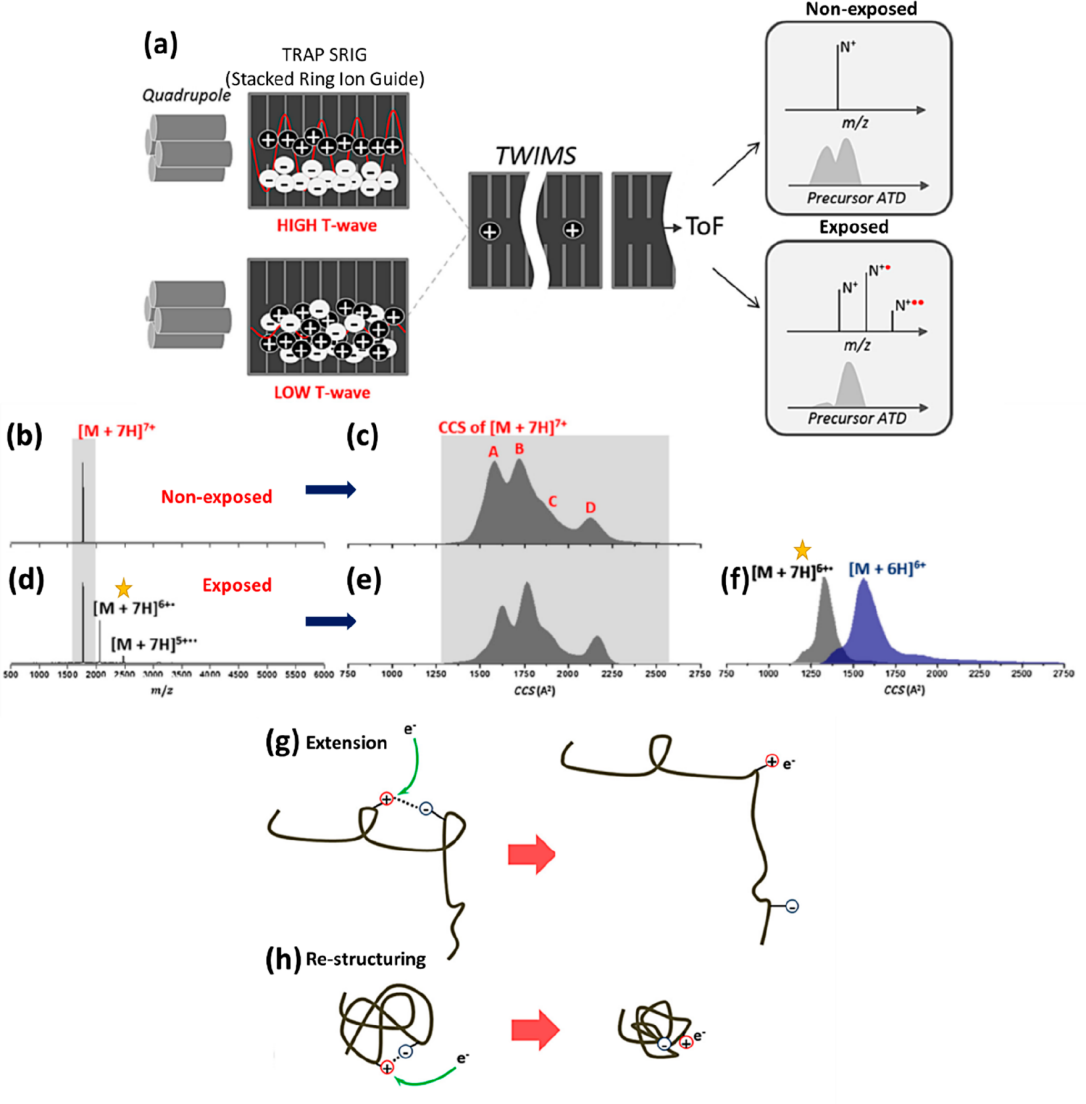
(a) Workflow of electron transfer with no dissociation ion mobility mass spectrometry (ETnoD IM-MS) on a traveling wave mass spectrometer using cytochrome c. The ETnoD reaction takes place in the trap region by lowering the wave height and allowing for the radical anions to interact with the mass selected (quadrupole) cations of interest. Both precursor and produce ions undergo mobility separation in the IM cell. Arrival time distributions and mass spectra are acquired and compared for both exposed and nonexposed ions to the ETnoD reaction. ATD are converted to collision cross section (CCS) distributions by calibration with proteins of known CCS. Mass spectra of the precursor cytochrome c ions which were not exposed (b) and exposed (d) to the radical anions of 1,3-dicyanobenzene. Two charge-reduced product ions produced [M+7H]^6+•^ and [M+7H] ^5+••^ (d). Collision cross sections (CCS) of the precursor [M+7H]^7+^ before (c) and after (e) exposure to the radical anions show coverage of almost similar conformational space (c,e) but decrease in intensities of the smaller conformers (A), narrowing of the distributions’ width in all conformers (A,B,D), and better resolving of conformer C after reaction with the ETD reagent (e). CCS comparisons between the first reduced product ion [M+7H]^6+•^ after the ETnoD reaction, noted with a star, and a [M+6H]^6+^ ion generated via electrospray ionization show indications of structural compaction with lower CCS values in the former. Illustration of the hypothesis for the structural changes observed on the proteins once an electron is transferred during the ETnoD reaction (g,h). The transfer on an electron disrupts the salt bridges (neutralizing contact) between opposite polarities, promoting the extension of the backbone chain which remains extended due to the electrostatic repulsion (g). The transfer on an electron disrupts the salt bridges (neutralizing contact) in a compact structure. If the free charged group pairs with an oppositely charged group in the surrounding area, restructuring in the form of compaction could be observed (h). Adapted with permission from ref (381). Copyright 2017 Elsevier BV.

IM-MS methods have been used to understand how the charge reduction of species impacts their conformation, stability, and dissociation pathways.^259,381,387,392−395^ In work by Jhingree et al., the conformation of proteins after charge reduction was investigated using electron transfer with no dissociation IM-MS (ETnoD IM-MS).^381^ As shown in Figure 23a, the trap stacked ring ion guide (SRIG) of a Synapt G2 Si is used to accumulate radical anions while in negative ion mode and cycled to allow these to react with positively charged analytes. The protein charge state of interest is mass selected in the quadrupole and also accumulated in the trap SRIG in positive ion mode. The wave height is then lowered, enabling the ETnoD reaction to occur. Both the charge-reduced (exposed) and precursor (nonexposed) are separated in the mobility cell, and the ATD are recorded and compared. For the charge reduction reaction, protein cations and radical anions from an electron transfer reagent (1,3-dicyanobenzene) were interacted in the trap region of the IM-MS instrument, prior to the IM separation, which was used to investigate conformational changes. In contrast to a conventional ETD experiment, where proteins also undergo dissociation, in ETnoD electrons are transferred from the reagent to the positively charged protein with no observable dissociation. The resulting ETnoD product are detected at reduced charge but with the same molecular weight as the parent ion.

From both standard proteins used, cytochrome C and myoglobin sprayed form both denaturing and native conditions, consistent decrease of the more compact precursor conformations intensity and narrower width was observed post exposure to the ETnoD reaction (Figure 23b–e). The narrowing was attributed, after experimental investigation, to collisional cooling of the ions in the trap SRIG cell. An exception found was that upon comparison between a [M+6H]^6+^cytochrome c ion formed in the ESI source under optimized conditions and a [M+7H]^6+•^ generated from the ETnoD charge reducing reaction, the latter was observed at lower CCS values, indicative of structural compaction (Figure 23f). For an ion of a given net charge, they observe a consistent preference in the CCS distributions favoring the more extended conformations after exposure to the ETnoD reduction reaction. The interesting remark is how a single electron addition can lead to such structural rearrangement for ions that are the most abundant during the ESI process (Figure 23c,e).

The authors also propose a model on how the electron transfer from the reagent to the salt bridges stabilizing the structure can explain the compaction and extension observations in the experimental findings (Figure 23g,h). When an electron interacted with a given salt bridge formed between an acid and basic group, it neutralized the contact. What was observed in their ETnoD experiments was that higher CCS values (extension) were found at higher charge states or lower CCS values (compaction) at lower charge states. For the higher CCS values (Figure 23g), they suggest that the electron interacts with the protonated basic residue, disrupts the stabilizing salt bridge, and removes the negatively charged acidic group leading to the extension. For the lower CCS values, it was suggested that the exact same electron transfer approach happens, but the salt bridge is weakened, which might lead to Coulombically driven conformational tightening. Some possible cases discussed is the possibility of compaction due to the negatively charged acidic groups in search of a protonated pair.

#### 4.5.4 Use of Cation-to-Anion Proton-Transfer Reactions Couple to IM-MS to Inspect the Protein Folding in the Gas Phase

**Figure 24:**
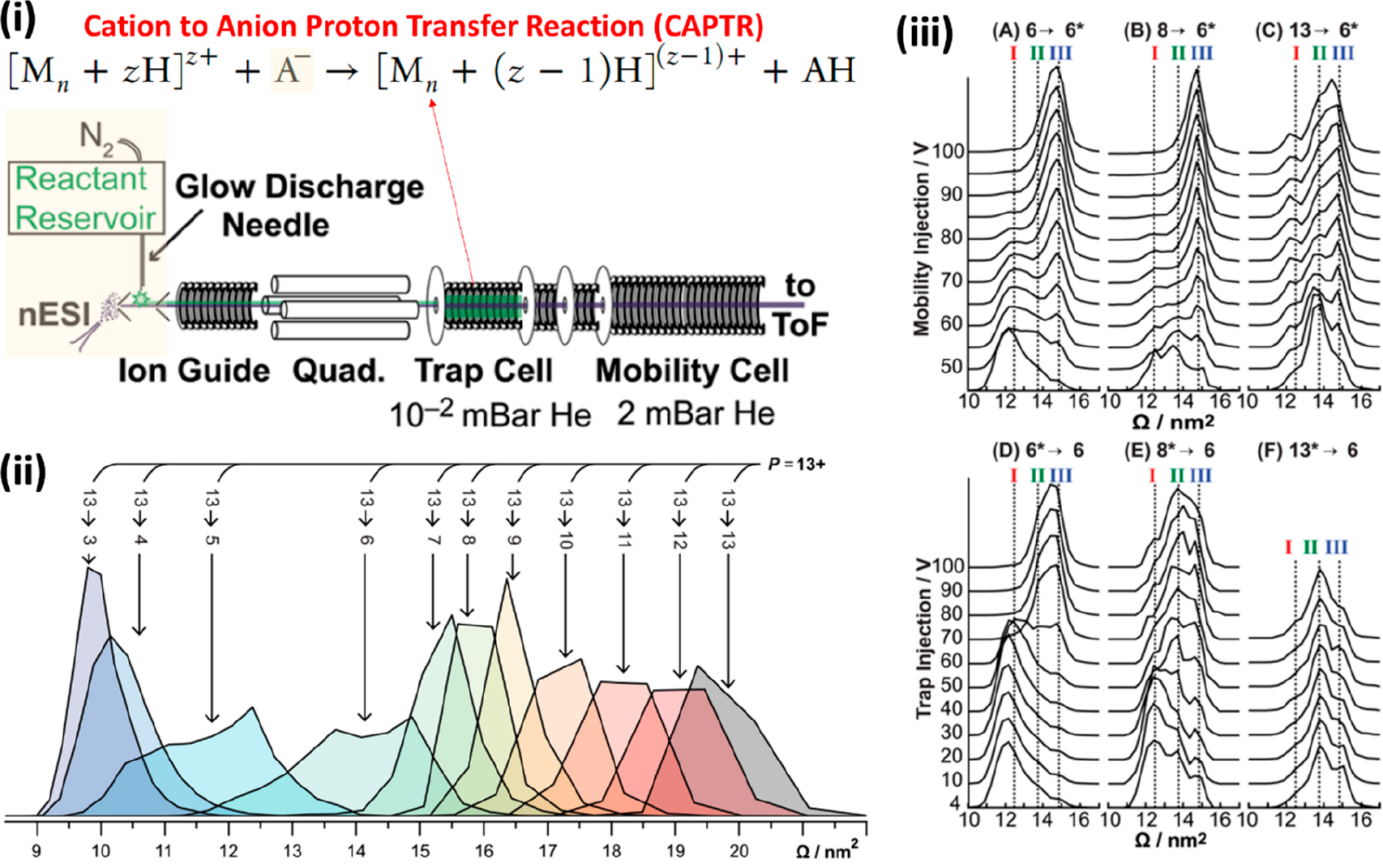
(i) A schematic of the modified quadrupole time-of-flight (Q-ToF) mass spectrometer (MS) used to perform the cation-to-anion proton transfer reaction (CAPTR) experiments, including the CAPTR reaction equation. Monoanions (A^–^) were generated via glow discharge (100 ms) of perfluoro-1,3-dimethylcyclohexane (PDCH), which leads to [PDCH-F]^−^ fragments with 381 *m*/*z*. The mass selected 381 *m*/*z* anions are accumulated in the trap cell. The instrument is then switched to +ve mode (2–3 s) and a charge state of the analyte of interest is quadrupole-selected and mixed with the [PDCH-F]^−^ in the trap cell for the initiation of the CAPTR reaction. The CAPTR products and nonreacted precursors are then separated in the IM cell. (ii) Normalized collision cross section (CCS) distributions for all 13+ ubiquitin (13→P) ions that underwent CAPTR. The products of CAPTR are noted as P → C, where P is the precursor ion charge state and C the product ion charge state. Any nonreacted precursor ions are shown as P → P. All ions exhibit monomodal CCS distributions except 13 → 6 (trimodal) and 14 → 5 (bimodal) CCS. (iii) Post (post-IM separation) CAPTR activation of (A) 6 → 6*, (B) 8 → 6*, and (C) 13 → 6* ions. Pre-CAPTR (pre-IM separation) activation of (D) 6* → 6, (E) 8* → 6, and (F) 13* → 6 ions. The asterisk indicates activation pre (P* → C) or post (P → C*) CAPTR. The vertical lines correspond to average CCS of three features (i–iii) from the CCS distributions of the +6 ion. Reproduced with permission from ref (387). Copyright 2016 American Chemical Society.

In similar work to Jhingree et al.,^381^ who used electron transfer to a positively charged protein ion,^396,397^ Kenneth et al. used cation-to-anion transfer reactions (CAPTR), proton transfer, to reduce the charge of denatured ubiquitin ions in the gas phase and probe their structure using native IM-MS and activated IM-MS before or after the reaction (Figure 24i). What they term as CAPTR can also be considered as an ion/ion proton-transfer reaction.^387^

In the CAPTR reaction noted as (P → C), a precursor charge state (P) is mass selected in the quadrupole and reacted with monoanions (A^–^) in the trap cell region to generate charge-reduced products (C).^398−401^ The monoanions were generated. Monoanions (A^–^) were generated via glow discharge (100 ms) of perfluoro-1,3-dimethylcyclohexane (PDCH), which leads to [PDCH-F]^−^ fragments with 381 *m*/*z*. The mass selected 381 *m*/*z* anions are accumulated in the trap cell. The instrument is then switched to positive ion mode for 2–3 s, and a charge state of the analyte of interest is mass-selected and mixed with the [PDCH-F]^− 387^. The CAPTR products and nonreacted precursors are then separated in the IM cell (Figure 24i).

A general observation from the native CAPTR IM-MS data was that all charged reduced ion exhibit a monomodal CCS distribution except the 13 → 6, which exhibits 3 features, and 13 → 5, which exhibits 2 features (Figure 24ii). Also, all P → 6 ions revealed three features (I–III), and their intensities are dependent on the precursor ion P. Comparing the CCS of the precursor and resulting charged reduced products, a general trend of lower CCS values was observed in the latter, indicative of structural compaction. The CCS values derived for the three features I, II, and III observed in the P → 6 ions were found to be larger than the same features in the native ubiquitin ion, of the same charge state. In contrast, for lower charge states, P → 3, the CCS value in both charge-reduced and native +3 ions were almost identical and in combination these findings demonstrate that CAPTR products adopt compact structures with decreasing charge state.

The CAPTR products were activated postreaction by increasing the voltage at which the ions entered the IM cell, to probe their structure and stability (Figure 24iii, A–C). It was observed that for all P → 6* ions, the CCS distributions do not significantly change after 85 V, they are independent of energy but heavily depend on the injection voltage below that threshold. Both 6 → 6* and 8 → 6* have high intensities of feature I until 85 V, and the former has higher intensities of features II and III compared to the latter (Figure 24iii). A contradicting observation is that in for 13→6*, feature I is persistent throughout the voltage range compared to 8 → 6* and 6 → 6*. The overall conclusion is that with the use of activation post the CAPTR reaction, they were able to suggest that the 6+ ion of ubiquitin presents at least two structures that are unable to interconvert with the current experimental conditions used. An alternative approach they took was to activate the precursor ions prior to CAPTR. This was done to establish whether the structure of the precursor affects the resulting charge-reduced products (Figure 24iii, D–F). For the 6 → 6* ion, increase in the trap injection voltage leads to depletion of feature I and promotion of feature III after 70 V (Figure 24iii, D), a similar trend observed for 6* → 6 ions as well (Figure 24iii, A).

Overall, they were able to show how one can use IM of CAPTR products to examine structural differences not obvious in more conventional IM. Both the CAPTR and the ETnoD studies are beautiful examples of how IM-MS can be combined with gas phase ion chemistry reactions to study the conformational landscapes of proteins in the gas phase.^387^

## 5 Conclusions and Outlook

From its roots in the study of measuring how atomic ions interact with gases, over the past 30 years, IM-MS has proven to be a versatile and informative analytical method for the study of the structure and dynamics of complex biomolecules. The incorporation of shape as a third separation dimension enables the study of biomolecules in multiple conformational populations in a single experiment. The CCS distributions of biological systems provide information on the conformational state of a given *m*/*z* selected species, which with careful tuning of solution and source conditions is reflective of the native form of the species under study. This method has particular advantages in the study of conformationally dynamic biological complexes where the CCS distributions can provide unique insights to lowly populated functional states. The fact that the mass spectrometer does not care if a protein is folded or natively disordered means this method has been shown to be extremely useful for examining IDPs or proteins with disordered and flexible regions. Once in the mass spectrometer, the ability to further probe conformational stability with collisional activation provides a unique and ideal laboratory from which to obtain robust and comparable data on the intrinsic fold and interactions of large biopolymers. With parallel work showing how it is possible to study protein complexes direct from crude expression lysates or even from endogenous sources including tissues, the power of this method to provide identity, stoichiometry, and structural information on low sample abundances, in situ, promises much for the future.

The ability to compare experimental CCS values with those obtained from other structural techniques or computationally derived structures means that IM-MS can be used to confirm or support atomistically refined conformers, and given low sample concentration requirements, this method has become a critical tool in initial evaluation of the conformational state of biological complexes. With technological advancements and the development of automated processing software^197^ and CCS predictive modes, the processing burden to make these comparisons is reducing, thus improving confidence in the data and allowing the researcher to study systems for which there is scant or no other structural information.

Challenges in the use of IM-MS to study biological complexes remain. We have highlighted the need to prepare samples carefully and encourage the use of ATD monitoring to monitor and prevent substantial conformational change as the complexes are transferred from solution into the mass spectrometer. Computational methods that can predict CCS values from candidate geometries are robust when helium is the drift gas but are less well trained on experimental data for other drift gases, even for nitrogen, the most ubiquitous in commercial instruments. The use of activation methods beyond collisional activation in collision cells, including on surfaces, with photons and electrons, and also with varying the temperature of the drift gas, are not routinely offered on commercial instruments, although we predict these will become more available in the coming years. Higher resolution IM-MS and the ability to perform tandem IM-MS experiments are exciting prospects in new instrumentation that allow better interrogation of complex structures as well as providing the ability to measure restructuring of biomolecules. Finally, we predict that combining IM-MS with soft landing of complexes for subsequent analysis with STM, or even cryo-EM will herald a new era of preparative structural biology, where conformationally and stoichiometrically selected species can be interrogated.

## Supporting Information Available

Supporting information to allow use of the toy model described in section 3.7.4 (XLSX)
